# Systems Biology of Coagulation Initiation: Kinetics of Thrombin Generation in Resting and Activated Human Blood

**DOI:** 10.1371/journal.pcbi.1000950

**Published:** 2010-09-30

**Authors:** Manash S. Chatterjee, William S. Denney, Huiyan Jing, Scott L. Diamond

**Affiliations:** Department of Chemical and Biomolecular Engineering, Institute for Medicine and Engineering, University of Pennsylvania. Philadelphia, Pennslyvania, United States of America; Medical College of Wisconsin, United States of America

## Abstract

Blood function defines bleeding and clotting risks and dictates approaches for clinical intervention. Independent of adding exogenous tissue factor (TF), human blood treated *in vitro* with corn trypsin inhibitor (CTI, to block Factor XIIa) will generate thrombin after an initiation time (*T_i_*) of 1 to 2 hours (depending on donor), while activation of platelets with the GPVI-activator convulxin reduces *T_i_* to ∼20 minutes. Since current kinetic models fail to generate thrombin in the absence of added TF, we implemented a Platelet-Plasma ODE model accounting for: the Hockin-Mann protease reaction network, thrombin-dependent display of platelet phosphatidylserine, VIIa function on activated platelets, XIIa and XIa generation and function, competitive thrombin substrates (fluorogenic detector and fibrinogen), and thrombin consumption during fibrin polymerization. The kinetic model consisting of 76 ordinary differential equations (76 species, 57 reactions, 105 kinetic parameters) predicted the clotting of resting and convulxin-activated human blood as well as predicted *T_i_* of human blood under 50 different initial conditions that titrated increasing levels of TF, Xa, Va, XIa, IXa, and VIIa. Experiments with combined anti-XI and anti-XII antibodies prevented thrombin production, demonstrating that a leak of XIIa past saturating amounts of CTI (and not “blood-borne TF” alone) was responsible for *in vitro* initiation without added TF. Clotting was not blocked by antibodies used individually against TF, VII/VIIa, P-selectin, GPIb, protein disulfide isomerase, cathepsin G, nor blocked by the ribosome inhibitor puromycin, the *Clk1* kinase inhibitor Tg003, or inhibited VIIa (VIIai). This is the first model to predict the observed behavior of CTI-treated human blood, either resting or stimulated with platelet activators. CTI-treated human blood will clot *in vitro* due to the combined activity of XIIa and XIa, a process enhanced by platelet activators and which proceeds in the absence of any evidence for kinetically significant blood borne tissue factor.

## Author Summary

Clotting of blood involves a series of reactions wherein at each step an inactive zymogen is converted to an active enzyme by the product of the previous step, sometimes in plasma and usually on efficient catalytic surfaces provided by the activating platelet. The protein Tissue Factor (TF) initiates this cascade when blood vessels are disrupted, but how this cascade is triggered in the absence of exogenous TF remains the subject of much debate. First, we validated a high throughput experimental system that allowed the noninvasive quantification of thrombin generation dynamics. Next, we showed that “contact activation,” despite use of the best available inhibitor (CTI) to prevent it, builds up enough autocatalytic strength to trigger coagulation without exogenous TF, particularly upon activated platelets. Further, we build an ODE based model to predict the stability of blood resulting from multiple perturbations with active enzymes at various physiologically realizable concentrations. Unlike existing models, we consider the dynamics of platelet activation on reaction rates due to phosphatiylserine exposure. The “Platelet-Plasma” model lays the groundwork for integration of coagulation reaction kinetics and donor specific descriptions of platelet function.

## Introduction

Blood coagulation is a platelet surface catalyzed protease cascade with autocatalytic amplification and multiple modes of inhibition [1]. Clotting *in vivo* is triggered by exposed tissue factor (TF) during vascular injury or plaque rupture. There is a striking sensitivity of coagulation to initial conditions of picomolar levels of TF [2]. Considerable uncertainty exists concerning the relative roles of circulating concentrations of TF [3], [4] and the mechanism(s) of platelet display of active TF via synthesis [4], [5], de-encryption [6] or changes in membrane composition [7]. The detection of circulating concentrations of activation peptides, such as F1.2 and fibrinopeptides A and B, inactivation products such as thrombin-antithrombin (TAT) [8], [9], [10], [11] and activated factors such as VIIa and activated Protein C (APC) is proof of some low level of activated proteases in normal individuals coexisting with flowing blood *in vivo*. Exploring this endogenous “engine-idling” active state of human blood *ex vivo* is central to clinical diagnostics. From a systems biology perspective that seeks to accommodate patient-specific variations, modeling can help address central questions regarding: (i) the regulation of response to perturbation, (ii) the stability of blood *in vivo* or *ex vivo* in the face of zero or near-zero levels of active TF or active proteases, and the (iii) safe pharmacological alteration of blood function.

Once initiated with TF, the coagulation cascade proceeds by sequential enzymatic steps to produce thrombin, which in turn polymerizes fibrinogen to fibrin to form the protein polymer mesh of the thrombus. Thrombin also has complex regulatory action by activating platelets and further enhancing thrombin formation by activating V, VII, VIII, and XI. *In vivo*, thrombin can limit its own production by binding thrombomodulin and converting protein C to activated protein C (APC), which subsequently inhibits Va and VIIIa. Upon activation, platelets express anionic phospholipids on their surface, an event that is critical for several surface bound reactions of the cascade [12].

Given the central role of thrombin in clotting, much effort has focused on experimentally detecting the transients of thrombin generation [13]. ‘Calibrated Automatic Thrombography’ measures thrombin formation by following the cleavage of more than 100 µM of the thrombin substrate Z-Gly-Gly-Arg-MCA [14]. However, the use of this fluorogenic substrate has been shown to interfere with the kinetics of the cascade by competitive inhibition of thrombin [15]. Furthermore, inhibitory influences of anticoagulants such as citrate may influence conclusions drawn from experiments with whole blood [16]. Complications due to artificial material activation of the contact pathway *in vitro* can be minimized by the inhibition of the contact system through the use of CTI to inhibit XIIa [17].

We have built upon the existing Hockin-Mann reaction network of the extrinsic coagulation cascade [18] to predict the function of blood studied *in vitro* with contact pathway inhibition. The Hockin-Mann model is a homogenous ordinary differential equation (ODE) reaction scheme that assumes a maximally activated platelet at *t* = 0. The Hockin-Mann model predicts accurately the initiation times of blood coagulation at picomolar levels of TF, but breaks down at zero or sub-picomolar levels of exogenously added TF [19]. Other models have attempted to consider the platelet as a separate entity which exists in discrete activation states and contributes binding sites once activated for the catalysis of several coagulation reactions [20], [21]. Such approaches have shed light on the critical importance of platelet activation mediated changes on the reaction surface area, the fragility of the cascade to thrombin induced activation of the platelet, stochastic effects at low molecular counts and experimentally observed variability in the initiation of clotting. However, these models still do not explain why blood drawn into CTI will clot without exogenous addition of TF.

Quantitatively, the systems biology analysis of blood clotting must account for strong autocatalytic feedback, nonlinearity of kinetic rates, and extreme sensitivity to initial conditions, specifically to help predict system outcomes that lead to insufficient or overactive responses (i.e. bleeding or thrombosis). The present study explores the stability of normal donor blood (i.e. what tips the balance towards clotting) as indicated by prolongation or reduction of the clotting time. We have developed and validated a high throughput assay to measure thrombin production initiation in static blood under minimal inhibitory influences of fluorogenic substrate and anticoagulant. We used this assay to examine several possibilities in existing literature that could account for initiation without exogenous TF and conclude that a leak of XIIa past saturating CTI is responsible for such initiation. Further, our studies demonstrated blood's graded sensitivity to exogenously added TF, IXa and XIa; a dramatic switch like response to exogenously added Xa; little discernible effect of exogenously added Va; and statistically significant VIIa activity (independent of TF) when used above nanomolar concentrations.

## Results

**Figure 1:**
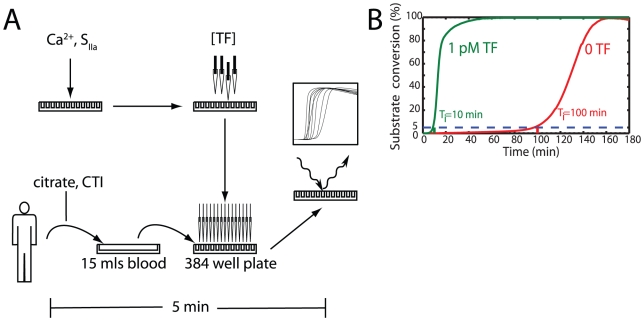
High throughput 384-well plate measurement of thrombin production in human blood. (**A**) **Experimental protocol.** Calcium and the fluorogenic thrombin substrate (S_IIa_) were added to the 384-well plate on a Thermo Multidrop. The plate was placed on a Perkin-Elmer Janus where various concentrations of each individual species were added to each well. After the blood was drawn, the plate was moved to a Perkin-Elmer Evolution P^3^ where the blood was added to all wells simultaneously (*t = 0*). The plate was read in a Thermo-Electron Fluoroskan where the fluorescence was measured for 4 hr. The time from vein to first measurement was under 5 min. (**B**) **Initiation Time.** The time required to reach 5% conversion of the fluorogenic substrate was set as the initiation time (*T*_i_). This metric correlated well with ∼10 nM TAT and preceded a burst of thrombin and a maximization of the second derivative of fluorecense. Relative prolongation or reductions in *T_i_* were used to quantify coagulation initiation.

We have used a high throughput fluorogenic assay to measure coagulation initiation in 384-well plates where 5×-diluted, citrated human blood (CTI treated) was recalcified within 5 min of venipuncture (**Figure 1A**). A level of 5% cleavage of the fluorogenic substrate Boc-VPR-MCA was used as the coagulation initiation time (*T_i_*). A large burst in thrombin production was always detected within minutes after *T_i_*. Relative prolongation or reductions in *T_i_* were used to quantify coagulation initiation in response to varying conditions (**Figure 1B**). The observed bursts in fluorescence were completely abolished by the thrombin inhibitor PPACK, and were not observed in prothrombin deficient (platelet supplemented) plasma (*not shown*), proving that we were indeed detecting thrombin and not some other non specific protease mediated cleavage of the fluorogenic substrate.

**Figure 2 pcbi-1000950-g002:**
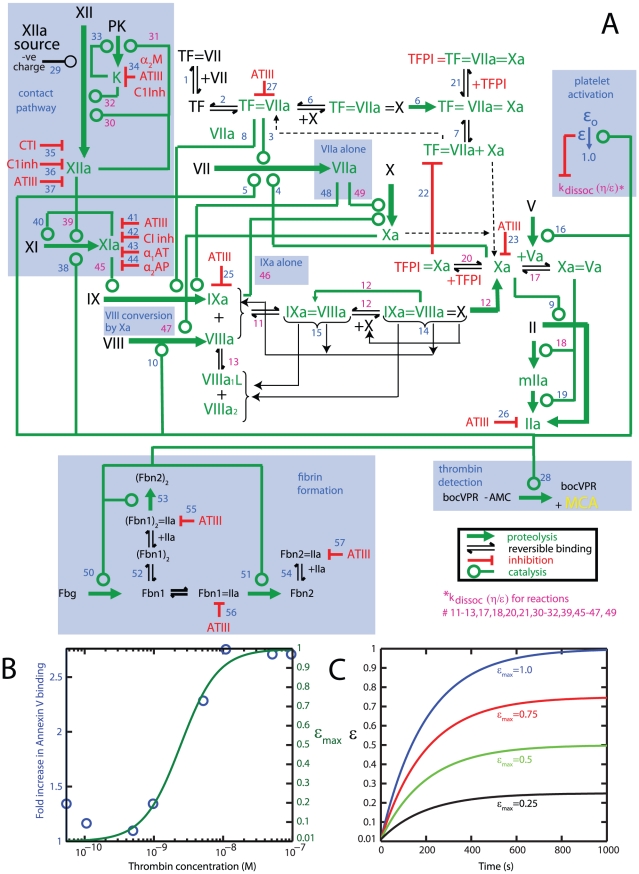
Schematic of the Platelet-Plasma model. (**A**) Wiring diagram of the Platelet-Plasma model. Blue highlighted portions represent additions to the Hockin-Mann model [18]. (**B**) Phosphatidylserine exposure measured by fold increase in annexin V binding was obtained from published values [60] and are shown in *blue circles*. The maximum platelet activation state attainable at a given thrombin concentration (ε_max_) was obtained by fitting a hill function to this data (*green line*). (**C**) The instantaneous platelet activation status (ε) approaches its maximum attainable value (ε_max_) on a time scale consistent with the time it takes for the platelet to mobilize intracellular calcium. Shown are ε transient profiles at various values of ε_max_. (*See text for complete mathematical descriptions of ε_max_ and ε*).

The Platelet-Plasma model (**Figure 2** and **Tables 1** **and** **2**) was built on the existing framework of the Hockin-Mann model [18] and our experimental observations. During the course of thrombin generation in the well plate, platelets become activated and this activation was modeled using a coarse-grained description of platelet function by equating platelet activation (ε) to phosphatidylserine (PS) exposure as a function of thrombin concentration (**Figures 2B and 2C**). The complete topology of the model, its constituent reactions, and initial conditions are shown in **Figure 2** and **Tables 1** **and** **2** (Also see **Supporting Text S1** for full listing of ODEs and reactions.) Justifications for making additions to the reaction topology of the Hockin-Mann structure are explained after the relevant experimental observations. Justifications for alterations to reaction kinetics are explained in the footnotes to **Table 1**.

**Table 1:** Reactions used in the Platelet-Plasma model.

Rxn	Model Expressions	k_1_	k_−1_	k_cat_ (s^−1^)	Hockin-Mann Model K_m_ or K_d_ (M)	η	Platelet-Plasma Model (ε = 1) K_m_ or K_d_ (M)	References
**1**	TF + VII ↔ TF = VII	3.2×10^6^ M^−1^ s^−1^	3.1×10^−3^ s^−1^		9.6×10^−10^	10	9.6×10^−9^	[61], [62]
**2**	TF + VIIa ↔ TF = VIIa	2.3×10^7^ M^−1^ s^−1^	3.1×10^−3^ s^−1^		1.3×10^−10^	0.01	1.3×10^−12^	[63]
**3**	TF = VIIa + VII → TF = VIIa + VIIa	4.4×10^5^ M^−1^ s^−1^						
**4**	Xa + VII → Xa + VIIa	1.3×10^7^ M^−1^ s^−1^						
**5**	IIa + VII → IIa + VIIa	2.3×10^4^ M^−1^ s^−1^						
**6**	TF = VIIa + X ↔ TF = VIIa = X → TF = VIIa = Xa	2.5×10^7^ M^−1^ s^−1^	1.05 s^−1^	6	2.8×10^−7^	0.01	2.4×10^−7^	[63], [64]
**7**	TF = VIIa + Xa ↔ TF = VIIa = Xa	2.2×10^7^ M^−1^ s^−1^	19 s^−1^		8.6×10^−7^	1	8.6×10^−7^	[65]
**8**	TF = VIIa + IX ↔ TF = VIIa = IX → TF = VIIa + IXa	1.0×10^7^ M^−1^ s^−1^	2.4 s^−1^	1.8	4.2×10^−7^	1	4.2×10^−7^	[66]
**9**	II + Xa → IIa + Xa	7.5×10^3^ M^−1^ s^−1^						
**10**	IIa + VIII → IIa + VIIIa	2.0×10^7^ M^−1^ s^−1^						
**11**	VIIIa + IXa ↔ IXa = VIIIa	1.0×10^7^ M^−1^ s^−1^	5.0×10^−3^ s^−1^		5.0×10^−10^	0.02	1.0×10^−11^	[67], [68]
**12**	IXa = VIIIa + X ↔ IXa = VIIIa = X → IXa = VIIIa + Xa	1.0×10^8^ M^−1^ s^−1^	1.0×10^−3^ s^−1^	8.2	8.2×10^−8^	0.01	8.2×10^−8^	[69]
**13**	VIIIa ↔ VIIIa_1_•L + VIIIa_2_	6.0×10^−3^ s^−1^	2.2×10^4^ M^−1^ s^−1^		2.7×10^−7^	0.01	2.7×10^−9^	[70], [71]
**14**	IXa = VIIIa = X → VIIIa_1_•L + VIIIa_2_ + X + IXa	1.0×10^−3^ s^−1^						
**15**	IXa = VIIIa → VIIIa_1_•L + VIIIa_2_ + IXa	1.0×10^−3^ s^−1^						
**16**	IIa + V → IIa + Va	2.0×10^7^ M^−1^ s^−1^						
**17**	Xa + Va ↔ Xa = Va	4.0×10^8^ M^−1^ s^−1^	0.2 s^−1^		5.0×10^−10^	0.04	2.0×10^−11^	[72]
**18**	Xa = Va + II ↔ Xa = Va = II → Xa = Va + mIIa	1.0×10^8^ M^−1^ s^−1^	103 s^−1^	63.5	1.6×10^−6^	0.02	6.5×10^−7^	[73]
**19**	Xa = Va + mIIa → Xa = Va + IIa	1.5×10^7^ M^−1^ s^−1^						
**20**	Xa + TFPI ↔ Xa = TFPI	9.0×10^5^ M^−1^ s^−1^	3.6×10^−4^ s^−1^		4.0×10^−10^	1	4.0×10^−10^	[74]
**21**	TF = VIIa = Xa + TFPI ↔ TF = VIIa = Xa = TFPI	3.2×10^8^ M^−1^ s^−1^	1.1×10^−4^ s^−1^		3.4×10^−13^	100	3.4×10^−11^	[64]
**22**	TF = VIIa + Xa = TFPI → TF = VIIa = Xa = TFPI	5.0×10^7^ M^−1^ s^−1^						
**23**	Xa + ATIII → Xa = ATIII	1.5×10^3^ M^−1^ s^−1^						
**24**	mIIa + ATIII → mIIa = ATIII	7.1×10^3^ M^−1^ s^−1^						
**25**	IXa + ATIII → IXa = ATIII	4.9×10^2^ M^−1^ s^−1^						
**26**	IIa + ATIII → IIa = ATIII	7.1×10^3^ M^−1^ s^−1^						
**27**	TF = VIIa + ATIII → TF = VIIa = ATIII	2.3×10^2^ M^−1^ s^−1^						
**28**	Boc-VPR-MCA + IIa ↔ Boc-VPR-MCA-IIa → Boc-VPR + AMC + IIa	1.0×10^8^ M^−1^ s^−1^	6.1×10^3^ s^−1^	53.8	-		6.1×10^−5^	
**29**	XII → XIIa	5.0×10^−4^ s^−1^						
**30**	XIIa + XII ↔ XIIa = XII → XIIa + XIIa	1.0×10^8^M^−1^ s^−1^	750 s^−1^	3.3×10^−2^	-	1	7.5×10^−6^	[75], [76]
**31**	XIIa + PK ↔ XIIa = PK → XIIa + K	1.0×10^8^M^−1^ s^−1^	3.6×10^3^	40	-	1	3.7×10^−5^	[75], [77]
**32**	XII + K ↔ XII = K → XIIa + K	1.0×10^8^M^−1^ s^−1^	45.3 s^−1^	5.7	-	1	5.1×10^−7^	[75], [78]
**33**	PK + K → K + K	2.7×10^4^M^−1^ s^−1^			-			[79]
**34**	K → K.Inhibited	1.1×10^−2^ s^−1^						[80]
**35**	XIIa + CTI ↔ XIIa = CTI	1.0×10^8^ M^−1^ s^−1^	2.4 s^−1^		-	-	2.4×10^−8^	[17]
**36**	XIIa + C1inh → XIIa = C1inh	3.6×10^3^ M^−1^ s^−1^						[81]
**37**	XIIa + ATIII → XIIa = ATIII	21.6 M^−1^ s^−1^						[81]
**38**	XI + IIa ↔ XI-IIa → XIa + IIa	1.0×10^8^ M^−1^ s^−1^	5 s^−1^	1.3×10^−4^	-	-	5.0×10^−8^	[82], [83]
**39**	XIIa + XI ↔ XIIa = XI → XIIa + XIa	1.0×10^8^ M^−1^ s^−1^	200 s^−1^	5.7×10^−4^	-	1	2.0×10^−6^	[78], [82]
**40**	XIa + XI ↔ XIa = XI → XIa + XIa	3.19×10^6^M^−1^ s^−1^						[46], [82]
**41**	XIa + ATIII → XIa = ATIII	3.2×10^2^ M^−1^ s^−1^						[84]
**42**	XIa + C1inh → XIa = C1inh	1.8×10^3^ M^−1^ s^−1^						[84]
**43**	XIa + α_1_AT → XIa = α_1_AT	1.0×10^2^ M^−1^ s^−1^						[84]
**44**	XIa + α_2_AP → XIa = α_2_AP	4.3×10^3^ M^−1^ s^−1^						[84]
**45**	XIa + IX ↔ XIa-IX → XIa + IXa	1.0×10^8^ M^−1^ s^−1^	41 s^−1^	7.7	-	1	4.9×10^−7^	[85], [86]
**46**	IXa + X ↔ IXa = X → IXa + Xa	1.0×10^8^M^−1^ s^−1^	0.64 s^−1^	7.0×10^−4^	-	1	6.4×10^−9^	[69], [87], [88]
**47**	Xa + VIII ↔ Xa = VIII → Xa + VIIIa	1.0×10^8^ M^−1^ s^−1^	2.1 s^−1^	0.023	-	1	2.1×10^−8^	[68], [89], [90]
**48**	VIIa + IX ↔ VIIa = IX → VIIa + IXa	1.0×10^8^ M^−1^ s^−1^	0.9 s^−1^	3.6×10^−5^	-		9×10^−9^	[91]
**49**	VIIa + X ↔ VIIa = X → VIIa + Xa	1.0×10^8^ M^−1^ s^−1^	210 s^−1^	1.6×10^−6^	-	1	2.1×10^−6^	[91]
**50**	Fbg + IIa ↔ Fbg = IIa → Fbn1 + IIa + FPA	1.0×10^8^ M^−1^ s^−1^	636 s^−1^	84	-		7.2×10^−6^	[47]
**51**	Fbn1 + IIa ↔ Fbn1 = IIa → Fbn2 + IIa + FPB	1.0×10^8^ M^−1^ s^−1^	742.6 s^−1^	7.4	-		7.5×10^−6^	[47]
**52**	2Fbn1 ↔ (Fbn1)_2_	1.0×10^6^ M^−1^ s^−1^	6.4×10^−2^ s^−1^					[47]
**53**	(Fbn1)_2_ + IIa ↔ (Fbn1)_2_ = IIa → (Fbn2)_2_ + IIa + FPB	1.0×10^8^ M^−1^ s^−1^	701 s^−1^	49	-		7.5×10^−6^	[47]
**54**	Fbn2 + IIa ↔ Fbn2 = IIa	1.0×10^8^ M^−1^ s^−1^	1.0×10^3^ s^−1^		-		1.0×10^−5^	[47]
**55**	(Fbn1)_2_ = IIa + ATIII → (Fbn1)_2_ = IIa: ATIII	1.6×10^4^ M^−1^ s^−1^						[47]
**56**	Fbn1 = IIa + ATIII → Fbn1 = IIa: ATIII	1.6×10^4^ M^−1^ s^−1^						[47]
**57**	Fbn2 = IIa + ATIII → Fbn2 = IIa: ATIII	1.0×10^4^ M^−1^ s^−1^						[47]

**Rxn 1.** Bach *et al.* [61] report a decrease in K_H_ from 14.9 to 0.58 nM as % phosphatidylserine (PS) increases from 0 to 40.

O'Brien *et al.* [62] report a k_assoc_ of 3.14×10^5^ M^−1^ s^−1^ and a k_diss_ of 6.29×10^−4^ s^−1^, which yields a K_d_ of ∼2 nM. With our choice of η, K_d_ is comparable to this value.

**Rxn 2.** Shaw *et al.* [63] report a decrease in K_d_ from ∼60 pM to ∼10 pM as % PS increases from 10–40. on TF liposomes and a decrease from ∼90 pM to ∼10 pM as % PS increases from 10–70 on TF nanodiscs.

**Rxn 6.** Shaw *et al.* [63] also report an ∼20× decrease in K_m_ from ∼400 nM to 20 nM for X activation as % PS increases from 10 to 40. Baugh *et al.* [64] report an experimental K_m_ of 238 nM on 25% PS vescicles.

**Rxn 7.** The product inhibition of TF∶VIIa by Xa is dependent on local Xa concentrations. Given that Xa and X binding to a PSPC bilayer increases hyperbolically with K_d_s of 53.9 and 34.2 nM respectively [65] and that the TF∶VIIa∶X complex is strengthened with increasing PS content (see 6), it is reasonable to assume that the TF∶VIIa∶Xa complex is also strengthened with increasing PS levels.

**Rxn 8.** Beals *et al.* [66] report that the K_d_ for bovine IX binding to lipid surface at optimum [Ca^2 + ^] decreases from 4.9 to 1.7 µM as % PS increases from 20 to 50. Given that the formation of TF∶VIIa is favoured with increasing PS content (See 2) and analogous to the increased strength of the TF∶VIIa∶X complex with increased PS (see 6), we expect that the formation of TF∶VIIa∶IX will also increase with increasing PS content.

**Rxn 11.** Jenkins *et al.* [67] report a decrease in K_d_ from 351 nM to 4 nM on PCPS vesicles. Neuenschander and Jesty [68] report a K_d_ of 74 pM on activated platelet surfaces as opposed to 550 pM on equimolar PSPC vesicles. A K_d_ of 10 pM on the activated platelet was required to fit the shape of the IXa titration in **Figure 9B**.

**Rxn 12.** Rawala Sheikh *et al.* [69] report a decrease in K_m_ from 45µM to 160nM from using unactivated to activated platelets respectively.

**Rxn 13.** Fay *et al.* [70] report a K_d_ value of ∼260 nM for this interaction at pH 7.4 in the absence of phospholipids. Fay *et al.* [71] report that this interaction is stabilized by the presence of phospholipid. In the Platelet-Plasma model this dissociation constant changes from 270 nM to 2.7 nM as the platelet activates.

**Rxn 17.** Lindhout *et al.* [72] report a decrease in K_d_ from 3.3 nM in solution to 30 pM using 10µM 40% PS.

**Rxn 18.** Rosing *et al.* [73] report a decrease in K_m_ from 34.5 to 0.21 µM using 7.5 µM phospholipids.

**Rxn 20.** Huang *et al.* [74] report a decrease in K*_i_* from 85.2 to 65.2 pM on using phospholipids.

**Rxn 21.** Given that the TF∶VIIa∶Xa (see 7) and the Xa∶TFPI (see 20) complexes are strengthened on phospholipids, we expect the stabilization of the TF∶VIIa∶Xa∶TFPI complex with the exposure of phospholipids as the platelet activates.

Baugh *et al.* [64] report an off rate 3.6×10^−4^ s^−1^ for Xa unbinding Xa∶TFPI, and on rates experimentally determined to be 9.0×10^5^ M^−1^s^−1^, or numerically estimated to range between 6.8×10^5^ and 1.35×10^6^ M^−1^s^−1^. Their data imply that these constants are comparable to those for the binding of TF∶VIIa∶Xa to TFPI (ie a K_d_ between 2.66×10^−10^ and 5.29×10^−10^ M). The original constants for this reaction in the Hockin-Mann model were fitted empirically, but their choice of constants results in a far stronger complex than can be reasonably expected from literature. Hence we have increased K_d_ by two orders (η = 100) of magnitude from their reported value.

**Rxn 28.** Experimentally determined by fitting initial velocities of AMC release to standard Michelis-menton kinetics.

**Rxn 29.**XII activation was coarse grained by assuming a first order dependence on XII concentration and estimating a rate of production (5×10^−4^s^−1^) that would resolve the disparity between the Hockin-Mann model prediction and the experimentally observed control.

**Rxn 30.** Kinetics of XIIa autoactivation (in the presence of negatively charged dextran sulfate) was from Tankersley *et al.* [75]. Griep *et al.* [76] showed that the autoactivation (and Kallikrein activation, See 32) of XII is strongly promoted by negatively charged sulfatides.

**Rxn 31.** Kinetics of Pre-Kallikrein activation by β-XIIa (in the presence of dextran sulfate) was from Tankersley *et al.* [75]. Pre-Kallikrein activation by XIIa was shown to be facilitated by negatively charged phosphoinositides [77].

**Rxn 32.** Kinetics of XII activation by Kallikrein (in the presence of dextran sulfate) was from Tankersley *et.al* [75]. Walsh and Griffin [78] showed that this reaction is sped up by the presence of activated platelets.

**Rxn 33.** Kinetics of second order Kallikrein autoactivation was from Tans *et al.* [79].

**Rxn 34.** The pseudo first order rate constant for the inhibition of Kallikrein in plasma (by C1 inhibitor, α_2_-macroglobulin and ATIII) was obtained from Van-Der-Graaf *et al.* [80].

**Rxn 35.** Hojima *et al.* [17] report a K_i_ of 24 nM for the inhibition of XIIa by CTI.

**Rxns 36 and 37.** Kinetics of XIIa inhibition by C1inhibitor and ATIII were from Pixley *et al.* [81]. C1 inhibitor is the primary inhibitor of XIIa. ATIII inhibition (although minor) was considering for consistency with other inhibitory reactions.

**Rxn 38.** Rate constants (in solution) for this reaction are from Gailani *et al.* [82]. Some controversy exists over the physiological surface for this reaction. Oliver *et al.* [83] showed that this reaction happens physiologically on the activated platelet surface. However several seminal papers by Baglia-Walsh *et al.* in the laboratory of Peter N. Walsh which originally proposed that this mechanism happens on the active platelet have subsequently been retracted. We therefore chose not to include a dependence of this reaction on ε.

**Rxn 39.** Rate constants (in solution) for this reaction are from Gailani *et al.* [82]. Walsh and Griffin [78], showed that this reaction is sped up by the presence of activated platelets.

**Rxn 40.** Several authors describe this mechanism of XI auto-activation (See for example [45], [82]). However, following the retraction of (Baglia *et al.* JBC 2000) we are not aware of an experimental report of the kinetics of this reaction. Kramoroff *et al.* [46]estimate the second order rate constant of this reaction to be 3.19 µM^−1^s^−1^ by optimizing an ODE model of the intrinsic cascade to experimental measurements of APTT. They consider either XI autoactivation or XI activation by thrombin (but not both possibilities) as plausible mechanisms for XI activation (in addition to activation by XIIa), thus their estimated value is likely an overestimate. We utilized a value 4 fold lower than the value they report for this constant, since we consider thrombin activation of XI in addition to autoactivation. This was in keeping with the experimental titration of XIa **(** **Figure 9C** **)** where we have noticed strong sensitivity to even minute amounts of XIa.

**Rxns 41–44.** Rate constants are for inhibition of XIa in plasma are from Wuillemein *et al.* [84].

**Rxn 45.** Rate constants (in solution) for this reaction are from Walsh *et al.* [85]. Gailani *et al.* [86] propose a mechanism by which this reaction could happen on the platelet surface facilitated by the dimeric form of factor XI.

**Rxn 46.** Rawala - Sheikh *et al.* [69] report a reduction in K_m_ from 45 µM to 390 nM from unactivated to thrombin activated platelets. In later publications from the same lab, Scandura and Walsh [87] report a K_m_ of 16nM and a k_cat_ of 5.1×10^−4^ for the activation of X by IXa alone on SFLLRN activated platelets in a model where platelet bound IXa interacts with zymogen X, and Wilkinson *et al.* [88] report a K_m_ of 6.4 nM and a k_cat_ of 7.0×10^−4^.

**Rxn 47.** Rate constants were obtained from Leipold *et al.* [89] using catalytic efficiencies reported in Lollar *et al.* [90]. Activation of VIII by Xa, unlike activation by thrombin (reaction 10) was reported to be markedly dependent on the presence of either phospholipid or active platelet surface [68].

**Rxn 48.** Rate constants for this reaction were from Komiyama *et al.* [91]. Unlike the activation of X by VIIa alone (see 49) the K_m_ for this reaction was reported to be relatively constant over a wide range of added PCPS concentrations, thus unlike other unbinding reactions in the model there was no dynamic change in reaction rate with platelet activation.

**Rxn 49.** Rate constants for this reaction were from Komiyama *et al.* [91]. The authors report a decrease in K_m_ from 1.48 to 0.25 µM with PCPS levels increasing from 1.4 to 21 µM.

**Rxns 50–57.** Kinetics of fibrin polymerization are taken from Naski *et al.* [47].

Reactions 1–27 comprise the original Hockin-Mann Model.

For reactions 1–27, parameter values were from the Hockin-Mann model when a reference is not cited.

Except for reaction 13, ε wherever applicable operates on the off rate usually defined as k_−1_. For reaction 13, ε operates on k_1_ which is the actual unbinding rate of the VIIIa complex. The notation for this reaction is kept consistent with its description in the Hockin-Mann Model.

On-Rates were assumed to be diffusion limited (with a *k_1_* of 1.0×10^8^ M^−1^ s^−1^) [20], and the corresponding off-rate was calculated from *K_m_* using 
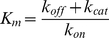
.

**Table 2:** Initial conditions of species in the Platelet-Plasma model.

Species	Hockin-Mann Model Initial Concentration (M)	Platelet-Plasma Model Initial Conditions (M)
TF	0	0
VII	1.0×10^−8^	1.0×10^−8^
TF = VII	0	0
VIIa	1.0×10^−10^	1.0×10^−10^
TF = VIIa	0	0
Xa	0	0
IIa	0	0
X	1.6×10^−7^	1.6×10^−7^
TF = VIIa = X	0	0
TF = VIIa = Xa	0	0
IX	9.0×10^−8^	9.0×10^−8^
TF = VIIa = IX	0	0
IXa	0	0
II	1.4×10^−6^	1.4×10^−6^
VIII	7.0×10^−10^	7.0×10^−10^
VIIIa	0	0
IXa = VIIIa	0	0
IXa = VIIIa = X	0	0
VIIIa1-L	0	0
VIIIa2	0	0
V	2.0×10^−8^	2.0×10^−8^
Va	0	0
Xa = Va	0	0
Xa = Va = II	0	0
mIIa	0	0
TFPI	2.5×10^−9^	2.5×10^−9^
Xa = TFPI	0	0
TF = VIIa = Xa = TFPI	0	0
ATIII	3.4×10^−6^	3.4×10^−6^
Xa = ATIII	0	0
mIIa = ATIII	0	0
IXa = ATIII	0	0
IIa = ATIII	0	0
TF = VIIa = ATIII	0	0
Boc-VPR-MCA	-	1.0×10^−5^
Boc-VPR-MCA = IIa	-	0
Boc-VPR	-	0
AMC	-	0
XII	-	3.4×10^−7^
XIIa	-	0
XIIa = XII	-	0
PK	-	4.5×10^−7^
XIIa = PK	-	0
K = XII	-	0
K	-	0
CTI	-	4.2×10^−6^
XIIa = CTI	-	0
C1inh	-	2.5×10^−6^
XIIa = C1inh	-	0
XIIa = ATIII	-	0
XI	-	3.1×10^−8^
XI = IIa	-	0
XIa	-	0
XIIa = XI	-	0
XIa = ATIII	-	0
XIa = C1inh	-	0
α_1_AT	-	4.5×10^−5^
α_2_AP	-	1.0×10^−6^
XIa = α_1_AT	-	0
XIa = α_2_AP	-	0
XIa = IX	-	0
IXa = X	-	0
Xa = VIII	-	0
VIIa = IX	-	0
VIIa = X	-	0
Fbg	-	9.0×10^−6^
Fbg = IIa	-	0
Fbn1	-	0
Fbn1 = IIa	-	0
(Fbn1)_2_	-	0
(Fbn1)_2_ = IIa	-	0
Fbn2	-	0
Fbn2 = IIa	-	0
(Fbn1)_2_ = IIa = ATIII	-	0
Fbn1 = IIa = ATIII	-	0
Fbn2 = IIa = ATIII	-	0

The initial conditions for the first 34 values in Table 2, were set to the values used in the Hockin Mann model [18]. The level of Boc-VPR-MCA was set to 10 µM (this is the level of fluorogenic substrate we use in our experiment). The level of CTI was set to 4.2 µM corresponding to the concentration of 50 µg/ml used in the phlebotomy syringe. XII, XI Pre-Kallikrein, C1-Inhibitor, α_1_AT, α_2_AP and Fibrinogen are set to their plasma concentrations [84], [92], [93].

### Validation of experimental methods to detect thrombin dynamics

Calibrated Automatic Thrombography (CAT) [14] measures the initiation, propagation and destruction phases of the thrombogram. In seeking to trace the complete profile, CAT uses a thrombin substrate Z-GGR-MCA that binds weakly (K_m_ = 305 µM) and that is consumed slowly (k_cat_ = 1.86 s^−1^) and requires high concentrations (416 µM) to quantify the complete profile. However, fluorogenic substrates can act as competing substrates for thrombin, thereby slowing down positive feedback. Z-GGR-MCA can also serve as a competitive substrate for Xa. The magnitude and importance of such inhibitory mechanisms of Z-GGR-MCA are still debated [15], [22]. Since thrombin has a strict requirement for proline in the P2 position [23], we compared Z-GGR-MCA with the highly selective thrombin substrate Boc-VPR-MCA (K_m_ = 61 µM, k_cat_ = 53.8 s^−1^) used at [S_IIa_] < K_m_ to detect coagulation initiation.

**Figure 3:**
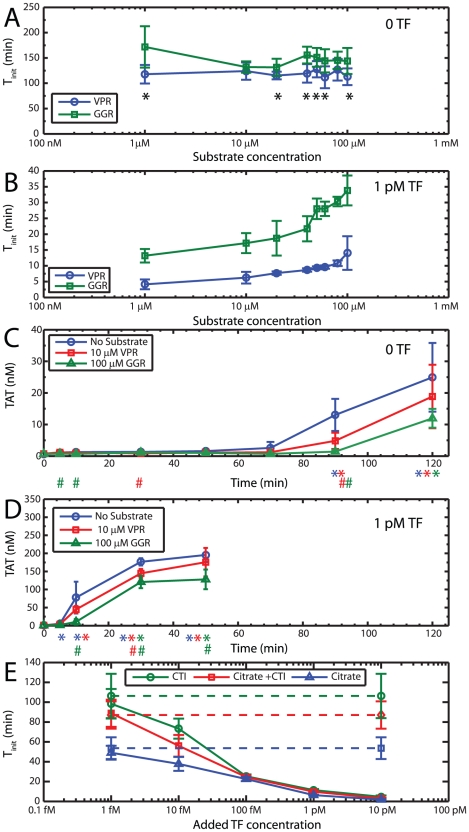
Validation of experimental protocol. (**A**) A titration of the fluorogenic substrates Boc-VPR-MCA *(blue circle)* and Z-GGR-MCA *(green square)* with 0 added TF showed a mild inhibitory influence of Z-GGR-MCA. *s indicates statistically significant difference (p<0.05) between initiation times detected with the two different substrates. (**B**) A titration of the fluorogenic substrates Boc-VPR-MCA and Z-GGR-MCA with 1 pM added TF showed inhibitory influence of both substrates where Boc-VPR-MCA was found to be the better substrate to detect initiation and exhibited little inhibition at 10 µM concentration. (**C**) TAT formation with 0 added TF, in the absence and presence of fluorogenic substrates showed less inhibitory influence of Boc-VPR-MCA on initiation defined by a burst in TAT compared to Z-GGR-MCA. Absolute [TAT] after initiation is decreased in the presence of either substrate. (**D**) TAT formation with 1 pM added TF, in the absence and presence of fluorogenic substrates showed decreased [TAT] during the propagation phase of coagulation in the presence of either substrate. Initiation detected by TAT correlated well with *T_i_* determined by our fluorogenic assay. In panels C and D, * indicates [TAT] significantly greater than baseline levels (p<0.05) and # indicates statistically significant differences compared to no substrate (*blue*). Experiments in panels A, B, C and D were carried out with blood from the same phlebotomy. (**E**) TF titration done in blood anticoagulated with CTI alone *(green)*, Citrate + CTI *(red)* and Citrate alone *(blue)*. Dashed lines indicate controls with no added TF. No significant difference was detected in titrations done with and without citrate, showing no evidence of inhibition by the anticoagulant. Effects of the contact factor pathway were apparent only below 100 fM added TF.

Titrations of the fluorogenic substrates Boc-VPR-MCA and Z-GGR-MCA were carried out at 0 and 1 pM added TF (**Figures 3A and 3B**) to determine the initiation time (*T_i_*). Since increasing substrate will take increasing time to be consumed, the 5% conversion criterion (*see Methods*) cannot be used as the metric to quantify coagulation initiation. We defined instead, the time when the second derivative of fluorescence was maximized as *T_i_* for this experiment. Without addition of TF (**Figure 3A**), the use of Z-GGR-MCA prolonged *T_i_* by 10 to 20 min at all concentrations from 1 to 100 µM in comparison to Boc-VPR-MCA. At 1 pM added TF (**Figure 3B**), both fluorogenic substrates were found to be inhibitory to some degree as demonstrated by a prolongation in *T_i_* with increasing substrate concentration. However, Z-GGR-MCA caused a prolongation of *T_i_* from 13.1 to 33.8 min as substrate concentration was increased from 1 to 100 µM, while Boc-VPR-MCA prolonged *T_i_* from 4.1 to 14.0 min as fluorogenic substrate concentration was increased from 1 to 100 µM. Thus, Boc-VPR-MCA used at up to 100 µM provided smaller *T_i_* (less inhibition) when compared to Z-GGR-MCA even when used at 1 µM.

Thrombin-antithrombin (TAT) formation provided an independent method to validate the fluorogenic assays (**Figures 3C and 3D**). Saturation of the detector (at maximized gain to detect low substrate concentrations) prevented the testing of 416 µM Z-GGR-MCA typical of CAT, thus the use of 100 µM Z-GGR-MCA in this comparison. In the absence of added TF (**Figure 3C**) clotting initiation was observed in ∼90 min as indicated by a change from baseline levels of TAT. The addition of 10 µM Boc-VPR-MCA or 100 µM Z-GGR-MCA slowed the formation of TAT with 0 or 1 pM TF addition compared to reactions without fluorogenic substrate (**Figures 3C and 3D**). In the presence of 1 pM TF (**Figure 3D**), clotting initiated at [TAT]∼10 nM at a time between 5 to 10 min in the absence of substrate or with 10 µM Boc-VPR-MCA present. However, clotting initated at [TAT]∼10 nM at a time between 10 to 30 min with 100 µM Z-GGR-MCA present. TAT formation in the propagation phase of thrombin production was inhibited by the presence of 100 µM Z-GGR-MCA substrate. Importantly, we have verified that [TAT] at the time of *T_i_* detected by the fluorogenic assay with 10 µM Boc-VPR-MCA was 10 to 20 nM which was in excellent agreement with the initiation phase of coagulation as defined by the time it takes to generate 10 nM thrombin [24].

The use of a more selective substrate Boc-VPR-MCA at a low concentration of 10 µM (used for the remainder of this study) allows for accurate detection of only the initiation phase and not the entire thrombogram, due to the rapid consumption of the substrate much before the complete conversion of prothrombin. However, the time taken for onset of initiation of thrombin generation is one of the most important characteristic of a thrombogram, and the use of the substrate at low levels allows for accurate detection of this metric without appreciable competitive inhibition of thrombin.

This was the basis for adding the thrombin detection chemistry (**Reaction 28**, **Table 1**) to the Hockin-Mann topology. In keeping with our experimental observation, thrombin formation is slightly inhibited by thrombin occupying the fluorogenic substrate and thus unavailable to generate positive feedback upon the zymogens V, VII, VIII, XI or activating the platelet.

The nonspecific effects of citrate [16] and CTI [25] were also evaluated by performing a titration of TF into blood drawn into CTI alone, CTI-citrate, or citrate alone (**Figure 3E**). Recalcification was done to adjust the concentrations of Ca^2+^ in each of these citrated blood samples to 10 mM. The time difference between recalcification of all the samples to the same [Ca^2+^] and the drawing of uncitrated blood (which was free to coagulate in the presence of physiological Ca^2+^ concentrations) was less than 3 min. It has recently been suggested that coagulation studies done with recalcified citrated blood are potentially influenced by inhibitory effects of calcium citrate [16]. Similarly, CTI has been reported to interfere with factor VIII coagulant activity [25]. We detect no significant difference in *T_i_* upon carrying out titrations of TF in blood anticoagulated with CTI alone or CTI-citrate, indicating little inhibitory influence of citrate on the initiation of coagulation. In the absence of added CTI, the effects of contact pathway-induced production of XIIa were apparent only below 100 fM added TF as suggested by a lowering of *T_i_* in the absence of CTI. It should be noted that citrated blood is maintained for <5 min prior to recalcification in the current study.

### Coagulation initiation without added TF

**Figure 4 pcbi-1000950-g004:**
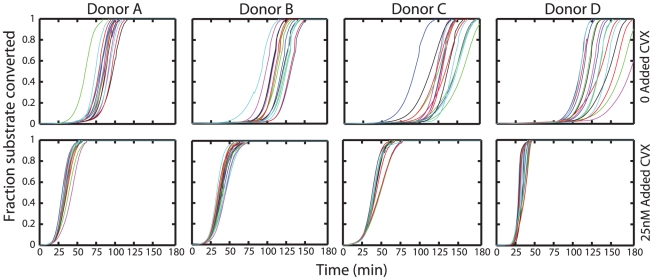
Coagulation initiation in the absence of externally added TF. Blood drawn into 50 µg/ml CTI and without added TF, will still reproducibly clot in ∼75 minutes. Prior activation of platelets with CVX will lower initiation time to ∼20 minutes. Shown are the multiple replicates tested under the same conditions with the same phlebotomy.

CTI additions beyond 32 µg/ml have been reported to saturate prolongations of clot times due to contact activation in otherwise unstimulated blood [1]. We reproducibly observe (**Figure 4**) that blood drawn into 50 µg/ml CTI will still clot between 60 to 120 minutes without addition of TF, for all donors tested. Prior activation of platelets with CVX will lower initiation time to ∼20 minutes. Similar results were obtained with other platelet agonists such as PAR1 peptide agonists or ADP (*data not shown*). Since contact activation is thought to be completely inhibited by the use of CTI, it was reasonable to hypothesize that TF from some “blood borne” source or other proximal triggers of prothrombinase formation were responsible for such initiation.

### Evaluating the role of ‘blood-borne’ TF and other mechanisms that could lead to initiation of clotting in blood drawn into CTI without exogenous TF addition

**Figure 5 pcbi-1000950-g005:**
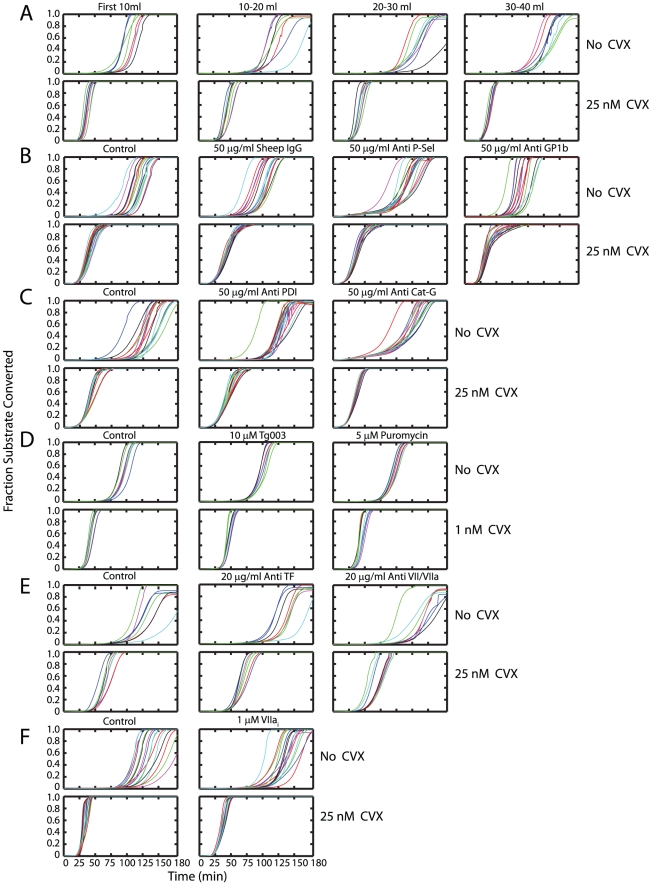
Evaluating mechanisms that could lead to initiation of clotting of blood drawn into CTI without exogenous TF addition. (**A**) To evaluate the effect of phlebotomy, experiments were conducted ±CVX using the first 10 mls, 10–20 mls, 20–30 mls and 30–40 mls of blood. No steady increase in *T*_i_ was noted showing that TF from phlebotomy was not leading to eventual initiation. (**B**) Addition of antibodies against P-selectin or Gp1b_α_ did not prolong initiation either in the absence or presence of high dose CVX, (**C**) Addition of antibodies against PDI or cathepsin G did not prolong initiation either in the absence or presence of high dose CVX. (**D**) The ribosome inhibitor puromycin; the *Clk1* kinase inhibitor Tg003; (**E**) antibodies against TF, VII/VIIa; or (**F**) VIIai did not prolong initiation either in the absence or presence of high dose CVX. This shows that initiation is unaffected by either ‘bloodborne’ or platelet synthesized TF on the time scales of our experiments.

To evaluate the effect of phlebotomy as a source of vessel wall derived TF, experiments were conducted with the first 10 ml, 10–20 ml, 20–30 ml and 30–40 ml of blood (**Figure 5A**). If TF was indeed produced from the phlebotomy, an increase in initiation time would be expected with subsequent volumes of blood drawn after the initiation of phlebotomy. Clotting initiated in the first 10 ml of resting blood in ∼75 min. For all subsequent volumes clotting initiated at ∼90 min and no further increase in initiation times was observed. Initiation occurred in 25 min in CVX pretreated blood for all volumes after the blood draw. The amount of TF due to phlebotomy appears minor and has no role in blood activated with CVX. In subsequent experiments, the first 10 ml of blood drawn was discarded.

P-selectin is expressed on the platelet surface upon activation and induces procoagulant PS exposure [26] and TF synthesis in monocytes [27]. TF-containing microparticles (MPs) have been reported to be generated by interactions between P-selectin and PSGL-1. Furthermore the presence of P-selectin on activated platelets helps to recruit these MPs to the developing thrombus [28]. However, the addition of a function-blocking P-selectin antibody caused no change in the time when a thrombin burst was detected, in the absence or presence of CVX (**Figure 5B**). VIIa can bind weakly to activated platelet surfaces and support thrombin generation independently of TF [29]. Weeterings *et al.* have reported recently that GP1bα can bind rFVIIa with a *K*_d_ of ∼20 nM and this interaction enhanced TF-independent thrombin generation [30]. However, neither antibodies against VII/VIIa (**Figure 5E**), nor antibodies against GP1bα (**Figure 5B**) had any effect on the initiation of thrombin formation with or without CVX stimulation. Protein disulfide isomerase (PDI) has been suggested as a possible trigger for tissue factor dependent fibrin generation. PDI originating from adhering platelets [31] can activate ‘encrypted or cryptic’ [6] functionally inactive TF by changing the disulfide status of the Cys186–Cys209 pair on the TF molecule [32]. Fibrin formation, following infusion of TF microparticles were shown to be strongly inhibited by an anti-PDI antibody *in vivo* [33]. However, in our hands an anti-PDI antibody had no significant effect in prolonging *T*_i_ with or without CVX treatment (**Figure 5C**), suggesting that mediated TF de-encryption via PDI was not a mechanism at play. This is consistent with earlier reports that: (1) exogenously added PDI or anti-PDI antibodies had no effect on TF-VIIa coagulant activity shown by MDA-MB231 cells stimulated with HgCl_2_; or (2) PDI silencing with PDI shRNA had no effect on procoagulant activity [34]. The integrin Mac-1 (CD11b/CD18) on neutrophils or monocytes can bind FX which can be converted to Xa by cathepsin G. Inflammatory stimuli or Mac-1 engagement of its ligands (including fibrinogen and FX) stimulates degranulation and release of cathepsin-G [35]. Such a mechanism of X activation could lead to initiation in the absence of TF. However, the addition of a anti-cathepsin G antibody had no effect in prolonging the time at which a burst in thrombin production occurred with or without CVX treatment (**Figure 5C**).

Platelets may have the capacity to rapidly splice TF pre-mRNA to a mature transcript and rapidly translate TF protein intracellularly [36]. We added Tg003 and puromycin to inhibit Cdc2-like kinase (Clk)1 and ribosomal function, respectively, two platelet functions reported to be responsible for splicing TF pre-mRNA and rapid TF mRNA translation [36]. A level of 10 µM Tg003 and 5 µM puromycin had no effect on the initiation time (**Figure 5D**), either in the absence or presence of high dose CVX. This suggests that the capacity of platelets to splice TF pre-mRNA and rapidly express TF protein does not contribute to clotting initiation at least on the time scales of the experiment. Also, the addition of 20 µg/ml anti-TF, 20 µg/ml anti-VII/VIIa (**Figure 5E**) or 1 µM active site inhibited factor VIIa, VIIa_i_ (**Figure 5F**) caused no increase in *T*_i_ with or without CVX activation of the platelets. This further suggests that in whole blood endogenous TF mediated mechanisms are not responsible for clotting initiation.

We note that in all experiments utilizing antibodies and without prior platelet activation there was slight reduction (relative to the control where no antibody was added) in the time taken for initiation to occur. For instance, sheep IgG by itself reduces T_i_ (**Figure 5B**) from 91.82±13.35 (control) to 70.08±10.57 min. Such effects were possibly mediated by non specific Fcγ-R mediated platelet activation [37] and were not apparent when the platelets were already fully activated with CVX.

### Evaluating the role of CTI

**Figure 6 pcbi-1000950-g006:**
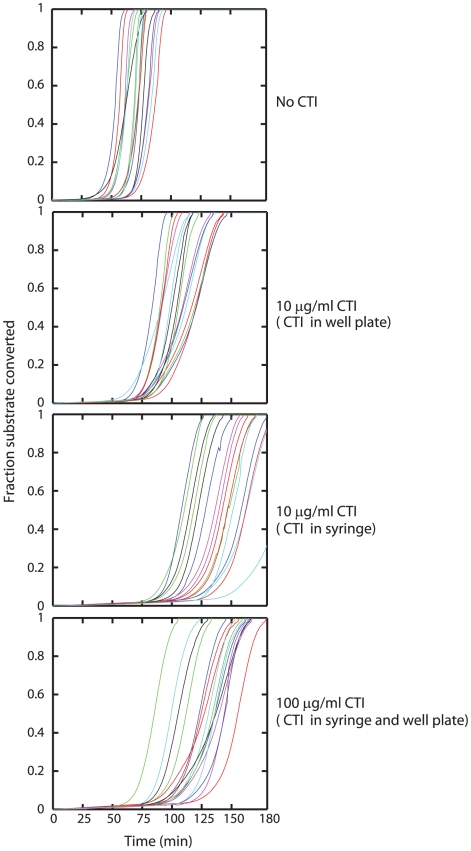
Saturation of the effects of CTI. To evaluate the possibility of leakage past CTI, experiments were conducted with no CTI, CTI addition in well plate, CTI addition during phlebotomy and large quantities of CTI during phlebotomy as well as in well plate. The inclusion of 50 µg CTI/ml whole blood (before a 5× dilution in the well plate) produced saturating effects.

In the absence of evidence for kinetically-significant blood borne TF, we evaluated the efficacy of high concentrations of CTI (**Figure 6**). Without inclusion of CTI in the syringe or in the well plate, clotting initiated in 57.3±9.9 min. When 10 µg/ml CTI (final concentration) was included in the well plate, initiation occurred in 77.4±7.0 min due to inhibition of XIIa production. When the same final concentration was achieved (after a 5× final dilution of blood in the well plate) by including 50 µg/ml CTI in the phlebotomy syringe, initiation time was markedly prolonged to 110.0±17.0 min. Liquid handling of citrated blood after phlebotomy takes ∼3 minutes before recalcification and this result points to the significant production of XIIa during this initial period. When very high concentrations (100 µg/ml) of CTI were achieved by including CTI in the syringe (50 µg/ml, diluted 5× in the well plate) and 90 µg/ml preexisting CTI in the well plate, initiation occurred in 96.6±15.1 min and was not prolonged further. Thus, the inhibitory effect of CTI saturates in spite of its use at very high concentration pointing to the possibility of a XIIa leak past CTI.

### Anti-XI and anti-XII used simultaneously prevent initiation in resting blood

**Figure 7 pcbi-1000950-g007:**
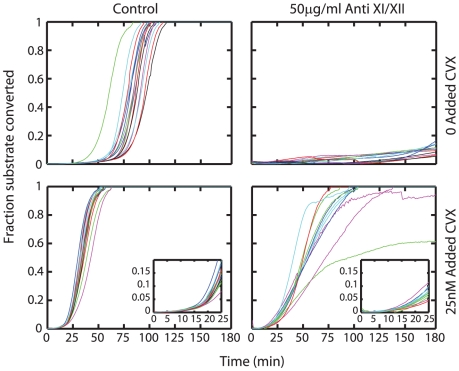
Effect of anti-XI and anti-XII. Addition of 50 µg/ml of anti-XI and anti-XII will completely prevent initiation of clotting in resting blood showing that initiation is a result of leak past saturated effects of CTI. However, on CVX activated platelets initiation is still unaffected by the presence of both CTI and these antibodies. Initial thrombin production during the propagation phase is however diminished due to abolition of thrombin feedback on FXI. Insert shows initial rates of thrombin formation in the presence of these antibodies.

Combined addition of 50 µg/ml of anti-XI and anti-XII to diluted whole blood (with CTI) completely abolished initiation (**Figure 7**) demonstrating that for conditions lacking platelet activation a steady leak of XIIa activity past CTI was the most proximal trigger of platelet activation and subsequent clotting of minimally perturbed blood *in vitro*. Interestingly, anti-XII when used alone did not prevent initiation (*not shown*), suggesting that inspite of our best attempts at preventing XII activation in diluted whole blood (CTI and anti-XII used together), some XIIa is still formed on the time scale of our experiments, necessitating the inhibition of XI activity as well. Also, anti-XI by itself did not prevent initiation in whole blood (*not shown*), suggesting strong autocatalytic amplification by very minute amounts of XIa formed inspite of the use of the inhibitory XI antibody. Despite the efficacy of high dose CTI in conjunction with anti-XI and anti-XII in preventing activation in resting blood, there was no effect of these antibodies on *T*_i_ when blood had been treated with CVX. The levels of thrombin produced during the initial moments of the propagation phase of thrombin production (after *T*_i_) were however diminished as demonstrated by lowered rates of fluorescence increase. This was possibly a consequence of the inhibition of thrombin- mediated feedback on FXI after initiation [38].

### Preventing initiation in CVX treated blood

**Figure 8:**
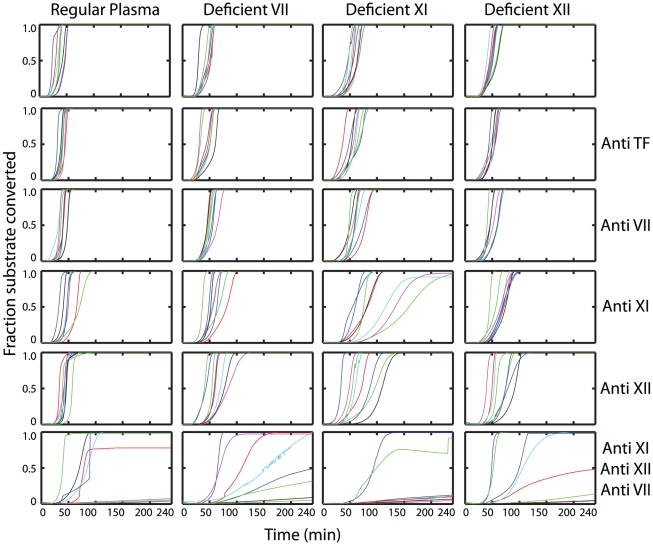
Prevention of initiation on CVX activated platelets. CTI-treated regular plasma or plasma deficient in factors VII, XI or XII were supplemented with washed (plasma free) platelets. These samples were left untreated or were treated with antibodies against TF; VII; XI; XII or XI, XII and VII simultaneously; and tested for thrombin generation without exogenous TF addition after activating platelets with 25 nM CVX. Simultaneous inhibition of XI, XII and VII activity was required to completely abolish thrombin generation.

Unlike the case of resting blood, initiation could not be prevented in blood pretreated with CVX by the use of antibodies against XI and XII. To probe further the mechanism responsible for such initiation, we supplemented CTI-treated regular plasma and CTI-treated plasmas deficient in factors VII, XI and XII with washed platelets. Thrombin generation in these samples as well as samples of plasma that had been treated with antibodies against TF, VII, XI, XII, or XI/XII/VII was studied following CVX activation **(** **Figure 8** **)**. Antibodies against TF had no detectable effect. Thrombin formation was not completely inhibited by blocking any single factor individually, but was inhibited in samples where the activities of XI, XII and VII were simultaneously inhibited (**Figure 8**, *last row of subplots*). In particular clotting was completely inhibited in 6 out of 8 wells in XI-deficient plasma treated with antibodies against XI, XII and VII. Inhibition of VIIa activity alone is insufficient (initiation times are almost unchanged in VII-deficient plasma treated with a VII antibody) because XIa formation beyond the XIIa leak past CTI, is still sufficient to trigger thrombin formation. Inhibition of XI activity (XI-deficient plasma with an XI antibody) has pronounced inhibitory effect, but was still insufficient with VIIa being functionally active on the active platelet. Thus upon activated platelets, in addition to blocking factors XI and XII, VIIa activity too must be inhibited to prevent thrombin generation.

It should be noted that immunodepleted plasmas deficient in coagulation factors have <1% normal activity of the particular factor, but are still not completely devoid of the depleted protein. This explains why initiation is prolonged further by antibodies against a protein that had already been immunodepleted in plasma. For instance thrombin generation in anti-XI treated, XI- deficient plasma is inhibited compared to that observed in XI-deficient plasma alone. The use of deficient plasmas offers a “cleaner inhibition” because the protein being targeted for inhibition is largely absent from the onset, as opposed to reliance on the inhibitory antibody to exert its effect before the protein is functionally active.

**Figure 9:**
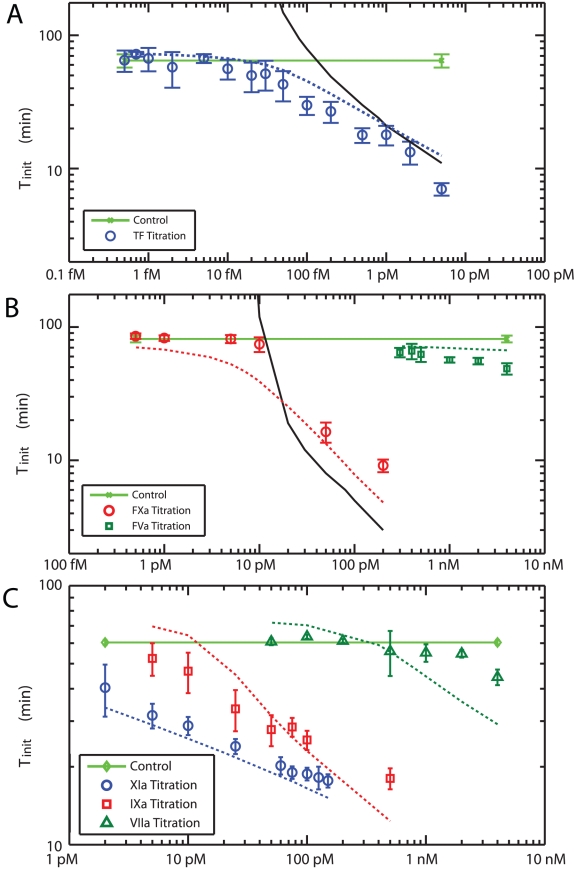
Titration of TF and active proteases into blood. (**A**) **Effect of exogenous TF on initiation time.** TF was titrated from 0.5 fM to 5 pM in 5× diluted blood. The black solid line is the simulated initiation time for the Hockin-Mann model (with S_IIa_) and the blue dashed line is the prediction of the Platelet-Plasma model. The light green solid line is the experimental control with no added TF. (**B**) **Addition of prothrombinase components.** Xa *(red)* and Va *(green)* was added to 5× diluted blood. The black solid line is the simulated initiation time for the Hockin-Mann model (with S_IIa_). The red and green dashed lines are the prediction of the Platelet-Plasma model for Xa and Va, respectively. The light green solid line is the experimental control with no added proteins. (**C**) **Addition of VIIa, IXa and XIa.** Various concentrations of VIIa *(green)*, IXa *(red)* and XIa *(blue)* were added to blood at 5× dilution. The dashed lines of the corresponding color are simulations done with the Platelet-Plasma model. The light green solid line is the experimental control.

Thrombin formation, once clotting is initiated by a single activator for instance by the addition of TF or Xa (*See* **Figure 9**) yields relatively small variations in initiation time when tested in multiple replicates. However, given the strongly autocatalytic nature of the cascade, complete abolition of a triggering mechanism is much more challenging. Even small localized diffusional or binding limitations of the inhibitory antibody will allow individual molecules that bypass the inhibitors to trigger the cascade. This accounts for the relatively large variations we observe in trying to inhibit initiation upon activated platelets, where thrombin formation occurs stochastically past inhibitory antibodies against targeted coagulation proteins.

### Titrations of TF and active proteases into blood

Addition of exogenous lipidated TF was used to initiate coagulation via the extrinsic pathway. Concentrations from 0.5 fM to 5 pM were added to whole blood **(** **Figure 9A** **)**. Added TF resulted in a continual, statistically significant reduction of initiation time over a wide dynamic range of 3 orders of magnitude of concentration from 20 fM to 20 pM. Distinct from flow experiments where a concentration threshold exists between 2 and 20 molecule-TF/µm^2^ [39], no switch-like regime was detected under static conditions. This suggests that the barrier to be overcome in the flow system to exceed the threshold is not a chemical inhibition barrier since such a barrier would be present under stasis as well. The observation is consistent with Fogelson and Tania [40] who note the barrier responsible for the threshold under flow is a physical one dependent on competition between flow-mediated removal and rate of production of procoagulant species.

Next, we investigated the individual components of prothrombinase (XaVa complex) on coagulation initiation **(** **Figure 9B** **)**. Addition of Va showed negligible effects with minimally decreased initiation time from 0.3 nM to 4 nM added Va (>10% of the plasma concentration of V). This relative insensitivity indicated that Va was not a limiting during coagulation initiation, in agreement with previous studies that show that Va may be present in platelets or be rapidly displayed or produced during activation [41]. In contrast, Xa additions produced a marked switch-like action by moving from a negligible change in *T_i_* at 10 pM to nearly saturating effect at 100 pM, a concentration that is <0.1% of the plasma concentration of X. This switch-like behavior was consistent with the well understood rate limiting role of Xa [18].

Further, we explored reactions proximal to thrombin production by addition of increasing amounts of Factors VIIa, IXa and XIa to blood **(** **Figure 9C** **)**. The nominal concentrations of endogenous VIIa (1% of VII) prior to exogenous addition in 5× blood is 20 pM [42]. Using 5× final blood dilution, we extended these concentrations to reach an effective concentration of VIIa above 1 nM. VIIa has no significant effect until above 1 nM, approximately 10% of the circulating amount of VII. For comparison, high dose rVIIa therapies use up to ∼17 nM final concentrations [43]. The dynamic range for added IXa extended from below 5 pM to above 500 pM and that for XIa extended from below 2 pM to above 200 pM. The upper end of these ranges are less than 1% of the circulating IX and XI concentration respectively [44]. Similar to results for TF, the addition of IXa or XIa in this assay did not display a switch-like regulatory function, i.e. changing from no effect to full effect over some narrow concentration range, but rather displayed a broad dynamic response over a wide range of concentrations.

Baseline low level protease activity can exist even in healthy individuals (the “engine idling” theory). Knowledge of the upper bounds of these proteases defines the levels of these enzymes that allow blood to remain in a stable state (flowing normally) in spite of their presence. Experimentally measured values of active proteases for a patient that are significantly different from these values might indicate underlying coagulation disorders or heightened sensitivity to weak triggers of clotting. Knowledge of the upper bound on proteases also is necessary for the accurate *in silico* modeling of human blood studied *in vitro*.

### Platelet-Plasma model of thrombin production in human blood

The Hockin-Mann model was used to simulate these tirations and was found to be quite accurate for TF concentrations above 1 pM, but diverged dramatically at concentrations below 1 pM (**Figure 9A**, *black solid line*) of added TF. We have shown experimentally, that a leak of XIIa past saturating levels of CTI is responsible for eventual intiation. This prompted us to include the contact factor pathway to Hockin-Mann topology.

Upon contact with anionic surfaces a conformational change occurs in the zymogen FXII, resulting in the formation of active FXIIa. The exact mechanism for XII surface adsorption, conformation change and activation remain ambiguous and cannot be modeled easily. We therefore coarse grained surface mediated XII activation by assuming a first order dependence on XII concentration (**Reaction 29**, **Table 1**) and estimating a rate of production (5×10^−4^s^−1^) that would resolve the disparity between the Hockin-Mann model prediction and the experimentally observed control *(green)* at 0 TF (without disrupting its predictive ability at high TF). It should be noted that this estimated rate of XIIa production was based solely on observations in our experimental surfaces (Corning 384-well plates) and may well be different on other surfaces. The small amount of XIIa that is initially formed auto-activates upon negative surfaces *in vitro* (**Reaction 30**, **Table 1**). Formed XIIa further activates Pre-Kallikrein to active Kallikrein (**Reaction 31**, **Table 1**), which in turn reciprocally activates XII (**Reaction 32**, **Table 1**) and Pre-Kallikrein (**Reaction 33**, **Table 1**). Both Kallikrein and XIIa are inhibited by plasma protease inhibitors (**Reaction 34**, **36–37**; **Table 1**). Further, XIIa is inactivated by CTI included at high concentrations specifically to inhibit contact activation (**Reaction 35**, **Table 1**). Despite the efficacy of CTI, it is a reversible inhibitor of XIIa; and thus there is always some active XIIa beyond CTI resulting from the unbinding of the XIIa = CTI complex. Such XIIa that has “leaked past” CTI can in turn activate XI (**Reaction 39**, **Table 1**). Thrombin feedback on XI has also been considered (**Reaction 38**, **Table 1**) as a mechanism for XI activation.

We have experimentally observed **(** **Figure 9C** **)** strong sensitivity of blood to very minute doses of XIa (even 2 pM of added XI reduced *T_i_* significantly from that of the control). Also complete abolition of clotting without exogenous TF, required inhibition of XI activity in addition to XII **(** **Figures 7** **and** **8** **)** suggesting that the very little XIa that is formed downstream of XIIa is able to strongly self amplify (especially if the pro-coagulant surface of the activated platelet is readily available). With inhibition by ATIII, C1-inhibitor, α_1_-antitrypsin and α_2_-antiplasmin (**Reaction 41–44;** **Table 1**), such sensitivity could not be adequately explained by just XIIa mediated activation and thrombin feedback on XI. Therefore we also included the mechanism of XI auto-activation [45] on negatively charged surfaces to the topology of the network (**Reaction 40**, **Table 1**). Following the retraction of Baglia and Walsh, JBC 2000 we are not aware of experimental reports on the kinetics of this reaction. However, Kramoroff *et.al* [46] estimate the second order rate constant of this reaction to be 3.19 µM^−1^s^−1^ by optimizing an ODE model of the intrinsic cascade to experimental measurements of APTT. They consider either XI autoactivation or XI activation by thrombin (but not both possibilities) as plausible mechanisms for XI activation (in addition to activation by XIIa), thus their estimated value is likely an overestimate. We utilized a value 4-fold lower than the value they report for this constant, since we consider both mechanisms.

The XIa that is formed activates IX (**Reaction 45**, **Table 1**). Unlike the original Hockin-Mann topology, IXa in the absence of its cofactor is now able to activate some factor X (**Reaction 46**, **Table 1**) albeit very inefficiently. This minute amount of Xa contributes to the activation of VIII (**Reaction 47**, **Table 1**), in addition to the VIII activation by thrombin in the Hockin-Mann structure (**Reaction 10**, **Table 1**). VIIIa once formed dramatically raises the efficacy of IXa and hence subsequent thrombin formation. Thus such participation of Xa in VIII activation (in addition to its ability to directly convert prothrombin) helps it to function in simulation as the dramatic switch like controller of the cascade we observed experimentally **(** **Figure 9B** **)**.

VIIa above 1 nM concentrations is able to produce significant lowering of *T_i_* independently of TF **(** **Figure 9C** **)**. Furthermore, with platelet activation, inhibition of VII activity was required (in addition to the inhibition of XI and XII activity) to completely block thrombin generation **(** **Figure 8** **)**. This suggests that VIIa activity independent of TF, is effective at high concentrations and on the activated platelet. This prompted us to include VIIa's ability to convert IX and X (independent of TF) in the structure of the model (**Reactions 48–49;** **Table 1**).

Fibrinogen can act as an efficient sink for thrombin in experiments conducted in whole blood, and thus limit its ability to generate positive feedback by converting other zymogens or activating platelets. The thrombin catalyzed formation of fibrin I and fibrin II, release of fibrinopeptides A and B and the irreversible entrapment of thrombin in complexes with fibrin and antithrombin are described in (**Reactions 50–57;** **Table 1**) using published kinetics [47].

With these additions to the structure of the Hockin-Mann topology, we could now obtain quantitative agreement between experimental and simulated titrations of the procoagulant proteins TF, Va, VIIa, IXa, Xa and XIa into 5× diluted blood. The original Hockin-Mann topology could predict finite *T_i_* for only high dose TF/Xa (*Solid lines in* **Figures 9A and B**).

Although there is evidence to suggest that TF dependent reactions **(** **Table 1** **, Reactions 1, 2, 6–8 and 21)** are sped up on acidic phospholipids (See Comments in footnotes to these reactions in **Table 1**), no ε dependence was used for these reactions in our simulations of the TF titration in **Figure 9A**. This is because TF was already added in lipidated form in these experiments, and thus the TF-VIIa assembly or activity will not be affected by changing lipid composition upon the platelet surface.

The Kuharsky-Fogelson model [20] for thrombin formation under flow in the presence of collagen activated platelets shows [Xa] remaining at levels of about 10 pM throughout the duration of simulation. In the Hockin-Mann model with a TF stimulus of 5 pM, Xa concentrations are 10–100 pM during the times that substantial thrombin formation first occurs and subsequently this rises by about 10-fold. Our results, along with the modeling predictions in these papers suggest why it is necessary that clotting be sensitive to low levels of Xa *in vivo*. Namely, if levels produced under flow (where Xa can be rapidly convected downstream) are to be efficacious at all, they must produce substantial effects at very low levels (∼10 pM), despite the presence of much higher concentrations of X, or the system's ability to produce much larger concentrations of Xa under conditions of stasis.

Similar to Kuharsky-Fogelson (Figure 7 of reference [20]), we show in **Figure S2 in Text S1** simulations for the concentrations of thrombin, Xa, Xa∶Va, and Va for a stimulus of 5 pM TF (Also, compare to Hockin-Mann, Figure 8B of reference [18]). We found free Xa concentrations growing to about 20 pM during the initial instants followed by a much more substantial burst (that increases Xa levels by ∼3 orders) following platelet activation. The Va concentrations grow steadily, but beyond full platelet activation (∼10 nM thrombin), there is a dramatic shift in the equilibrium towards Xa∶Va and thus the amount of free Va drops rapidly, as prothrombinase levels rise steeply.

### Simulations of clotting times in whole blood

Our high throughput experimental system allows us to efficiently study coagulation reactions under various perturbations with multiple replicates. However, reaction volumes in our 384-well plate systems are only 50 µl, preventing us from studying undiluted whole blood. Automated liquid handling can dispense with high precision volumes of 5 µl, but for lower volumes experimental variability is high. Since each individual well must contain several reagents (calcium, fluorogenic substrate, platelet agonists, antibodies and coagulation proteases) other than human blood, there is a limitation to the volume of blood that can be used in each well.

De Smedt *et al.* [48] have recently reported that anticoagulant pathways (particularly the TFPI and APC mediated) are more affected by dilution than the procoagulant pathways. However, they did not observe any significant effect of dilution on the duration of the initiation phase, both for TF and Kaolin stimulated coagulation reactions in PPP upto dilutions of 12× (detectable effects on endogenous thrombin potential and peak height of the thrombograms were however reported). Blood at 5× final dilution is below this limit of 12× dilution beyond which linear scaling of initial conditions with the dilution factor might not be sufficient to account for the kinetics of thrombin generation initiation, and provides a tractable system for extensive high throughput experimentation. In simulating such experiments, the initial conditions of all species were divided by 5 to account for the dilution in each well.

**Figure 10 pcbi-1000950-g010:**
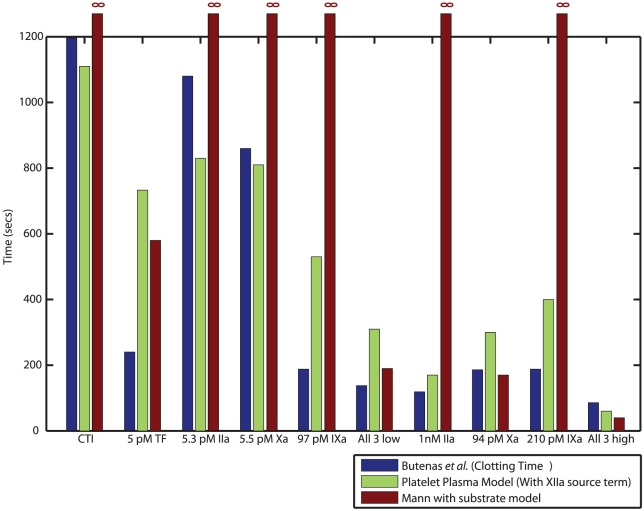
Simulating clotting times in whole blood. The very small reaction volumes in a 384 well plate prevent us from studying coagulation reactions in whole blood (*See text*). To simulate the kinetics of initiation in whole blood we simulated clotting times for additions of TF, thrombin (IIa), IXa, Xa or combinations of all 3 proteases at low and high doses reported by Butenas *et al.* [49] in the Mann laboratory. We found good qualitative agreement between experimental clotting times *(blue)* and initiation times predicted by the Platelet-Plasma model *(green)*. The Hockin-Mann (with fluorogenic thrombin substrate, S_IIa_) model (*red*) predicts finite initiation times only in the presence of high dose TF or Xa.

To test the ability of the model to predict perturbations in whole blood, we simulated clotting times (a good measure of the time to generate a burst in thrombin) for additions of either TF; IXa, Xa, thrombin; or all 3 proteases together reported by Butenas *et al.* [49]. We found good agreement between experimentally reported values of clotting time and predicted initiation times across all conditions **(** **Figure 10** **)**. The original Hockin-Mann model could predict finite *T_i_* in the presence of high dose TF/Xa, or when low dose thrombin, IXa and Xa were present simultaneously.

### Simulating platelet activation

The Hockin–Mann model assumes a fully activated platelet at *t = 0*. In the Kuharsky-Fogelson model [20] or the Cellular Potts model [50], platelets can exist either in discrete “unactivated” or “activated” states. We do not consider platelets explicitly, but all reaction rates reported in literature to be dependent on platelet activation are altered by a single function (ε) of thrombin concentration *(See Methods)*. As the platelets are activated by thrombin, they expose anionic phospholipids like PS on their surface, and the “platelet activation status” ε increases from a basal state of 0.01 to 1.00. The unbinding rates of all enzyme-substrate complexes are set to their literature values at saturating PS levels, but are all divided by ε. Thus, as the platelet activates the unbinding rates dynamically decrease by two orders of magnitude, consistent with the notion that the platelet surface becomes more procoagulant. In the Kuharsky-Fogelson model of intravascular thrombosis under flow, reaction rates depend on the availability of fully activated platelets (contributing maximal platelet binding sites for the coagulation factors). Since binding sites change with the concentration of activated platelets, the local effective reaction rates change as well. For modeling isotropic coagulation in a well plate, we account for a continuum of platelet activation states by thrombin and assume an excess of PS once platelets fully activate (as opposed to discrete saturable binding sites in the Kuharsky-Fogelson model).

Reactions that have been reported in literature (See **Table 1** footnotes) to be accelerated by acidic phospholipids are upregulated in the model when platelets are activated by thrombin as ε increases (and dissociation rates decrease). Platelets provide the negative surfaces for coagulation factor assembly *in vivo*, yet most current enzymology studies of coagulation enzyme kinetics *in vitro* use artificial phospholipid surfaces since standardizing platelet surfaces for kinetic assays is difficult.

In reality, surface adsorption of procoagulant molecules might also alter the catalytic efficiency (k_cat_), but for the sake of simplicity all k_cat_ values are set to their reported values at saturating PS levels (with the assumption that loose enzyme substrate complexes will hardly be formed in the first place and thus rarely get converted). The assumption that all unbinding rates change by two orders of magnitude is also made for simplicity. (In reality some enzyme substrate complexes are dramatically strengthened on exposed PS surfaces, while others show only weak dependence. *See* **Table 1** *footnotes*.)

We make the assumption that the amount of active surface available upon activation of platelets at normal platelet counts (3×10^6^ platelets per 50 µl reaction well for 5×diluted blood with a platelet count 3×10^8^ platelets/ml) is sufficient for coagulation reactions to proceed at their most optimal rates. Thus, like the Hockin-Mann model we assume an excess of phospholipid surfaces once the platelets are activated. Yet, our approach accounts for the fact that surface reactions occur at a rate dependent on the extent of platelet activation rather than being fixed at the highest level at t = 0.

As a pseudohomogeneous and single phase model of coagulation, we do not treat platelets as separate entities (or account for free and plate bound species). Thus, the fact that each platelet may have a limited number of specific binding sites is not considered. Our approach assumes that sufficient binding sites are available in 5×diluted blood, an assumption that may not be valid at greater dilutions. Also, we assume that coagulation in a well plate is well mixed, lacks spatial gradients, and is not transport-controlled. In contrast, intravascular thrombosis occurs under conditions with (1) substantial gradients of reactive species and platelet binding sites, (2) significant effects of convective transport on soluble species, and (3) intrathrombic diffusion limitations.

**Figure 11:**
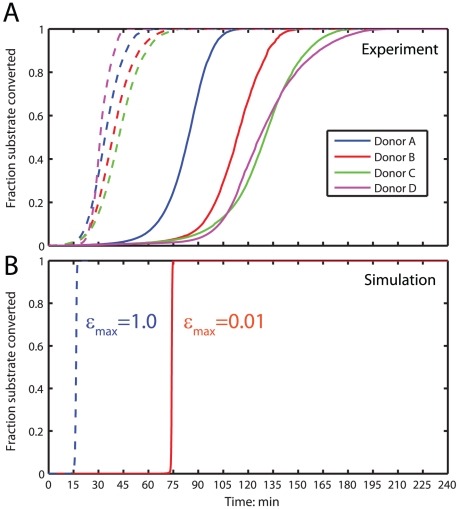
Simulating platelet activation. (**A**) Mean substrate conversion across all replicates for the donors shown in **Figure 4**. Substrate conversion traces without platelet activation are shown in solid lines and conversion traces upon activated platelets are shown in dashed lines. (**B**) Setting the initial activation state 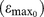 allows us to simulate platelet activation and its dynamic effect on all platelet dependent unbinding rates *(see Methods)*. The red line indicates simulations of substrate conversion without prior platelet activation 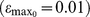. The blue dashed line indicate simulations of substrate conversion upon instantaneously fully activated platelets at *t = 0*. 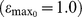.

Setting the initial activation state 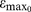, allows us to control in simulation the basal level of activation of the platelet **(** **Figure 11B** **)**. For 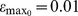 (akin to assuming 1% PS exposure on the surface of unactivated platelets), we obtain a burst in thrombin production (and hence fluorogenic substrate cleavage) at ∼75 min in the Platelet-Plasma model. To simulate experiments where coagulation reactions occur upon pre-activated platelets, 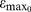 is set to a value of 1.0 (with the assumption that the fully activated platelets have exposed all of their PS) giving *T_i_*∼15 min. These values are in good agreement **(** **Figure 11A** **)** with the mean initiation times obtained upon unactivated platelets *(solid lines)* and activated platelets *(dashed lines)* for the 4 donors shown in **Figure 4**.

We note that the rate of thrombin production (substrate conversion) in simulation beyond *T_i_* increases more sharply than actually observed in experiment. Thrombin concentrations at *T_i_* are ∼10 nM, corresponding to a fully activated platelet **(** **Figure 2B** **)** beyond *T_i_*. This suggests that a method of prothrombinase inhibition (that has not been considered in our reaction topology) may exist once platelets are activated. Inhibition of XaVa *in vitro* in the absence of endothelial thrombomodulin is poorly understood. Further experiments will be required to pinpoint this inhibitory mechanism, but for the present the Platelet-Plasma model is a reliable indicator of the time taken to generate a burst in thrombin *in vitro*.

## Discussion

Human blood is the only living tissue that is routinely and easily obtained for *in vitro* research and clinical diagnostics. Blood is fully amenable to high throughput functional phenotyping and strong genotype-phenotype linkages are the basis of significant human disease. The platelet and the plasma form the basis for hemostasis and have been modeled from a bottom-up systems biology approach [18], [20], [21], [51] with the distinct advantage of relatively well defined reaction topology and freedom from genome-wide transcriptional complexity.

We have developed a high throughput assay that allowed us to study thrombin generation in diluted blood, across several conditions simultaneously with multiple replicates at each condition. The use of diluted blood in this assay is a limitation of the small reaction volumes in a 384-well plate and scales the initial concentrations of all species by the final dilution factor. The presence of an externally added fluorogenic substrate for the purpose of detection represents a subtle alteration of thrombin-mediated feedback of its own generation by competitively occupying thrombin's active site. We evaluated the commonly used thrombin substrates Z-GGR-MCA and Boc-VPR-MCA **(** **Figures 3 A, B, C and D** **)**. The latter substrate was found to have acceptably small inhibitory effects on *T_i_* at a concentration of 10 µM. In contrast, Z-GGR-MCA at a concentration of 100 µM was found to markedly hinder *T_i_* for 1 pM TF triggered coagulation. The use of 416 µM of this substrate in the ‘Calibrated Automatic Thrombography’ assay may be necessitated by the objective of tracing the ‘complete’ thrombogram. The use of 10 µM Boc-VPR-MCA suffices for the purpose of detecting coagulation ‘initiation’ and reduces assay interference. Also, the use of citrate as an anticoagulant was not found to have any significant influence on clot initiation times and permitted us to have a well defined activation time (*t = 0*) for the coagulation reaction space defined as the time of recalcification of all the wells. Effects of the contact factor pathway were significant at concentrations less than 100 fM TF and were subsequently prevented by the use of CTI (**Figure. 3E**).

We and others have consistently observed that CTI-treated human blood clots in the absence of added TF *in vitro*. In keeping with the notion of “blood borne TF” it is tempting to attribute such clotting initiation to platelet-dependent sources [5], [36]. We explored several possible mechanisms with neutralizing antibodies against TF, VII/VIIa, PDI, P-selectin, GPIb, and cathepsin G but none had affect on thrombin generation. Similar to the experiments of Butenas *et al.* [52], we have not found any evidence for functional TF in static whole blood (a closed system).

CTI inhibits β XIIa [53] and has relative specificity for this form of XIIa [54]. The β form of XIIa does not bind to surfaces and has little ability to activate XI, although it does activate Pre-Kallikrein (which in turn amplifies XIIa production). The effectiveness of CTI in prolonging APTT is probably a result of prevention of XIIa amplification by Kallikrein produced by β XIIa. The surface bound α form of XIIa is the main activator of FXI and the intrinsic coagulation system [55]. Such XIa produced by α XIIa on negative surfaces could get past CTI and lead to contact activation. We have demonstrated that in resting whole blood XIIa can leak past even very high concentrations of CTI, eventually leading to a significant burst in thrombin production **(** **Figure 6** **)**. Without CVX activation, the combined use of CTI, anti-XII and anti-XI block all proximal triggers of clotting in the 4 hr assay **(** **Figure 7** **)**. With CVX activation, VIIa activity on the active platelet alone (independent of TF) is kinetically significant, but the local activity of single molecules of XIa (formed downstream of a XIIa leak past CTI) in the proximity of the active platelet surface, can lead to efficient thrombin production **(** **Figure 8** **)**.

We highlight a recent report by Back-Nilsson *et al.* [56] where the authors showed that the levels of XIIa-AT, XIa-AT and Kallikrein-AT complexes were not affected by CTI treatment if contact activation of XII is allowed to occur on activated platelet surfaces. In contrast, if contact activation is allowed to occur on artificial negative surfaces like glass or kaolin, the formation of XIIa inactivation complexes is completely abolished by CTI (Back *et al.* Figures 1–3). Thus, there exists strong evidence that CTI is ineffective against XIIa formed on active platelet surfaces. Several reports attribute *in vitro* thrombin generation following platelet activation to endogenous TF. Caution must be exercised in interpreting such results because contact activation contact activation still occurs upon active platelet surfaces even in the presence of CTI (and antibodies against XII and XI).

Initiation times obtained by titrations of active proteases provide estimates of upper limits for endogenous active proteases. Activation peptides of several proteases have indeed been detected in blood indicating that these proteases do exist in active form albeit in concentrations low enough to have little effect on physiologic time scales [8], [9], [10], [11]. Our experimental titrations **(** **Figure 9** **)** bounded the initial conditions of active proteases (or TF) in blood to: <∼500pM Va, <1 nM VIIa, <1 pM IXa, <10 pM Xa, <1 pM XIa and <∼40 fM of pre-synthesized TF. Titrations of added thrombin are difficult to deconvolute using fluorogenic thrombin substrates. Circulating thrombin levels must be <100 pM to maintain platelets in an unactivated state **(** **Figure 2B** **)**. With respect to initial conditions of active proteases at *t = 0*, we attempted to search the initial condition space of blood using Simulated-Annealing and Particle-Swarm optimization algorithms to find the best fit of initial conditions, subject to the above upper bounds for non-zero initial levels of Va, VIIa, IXa, Xa, XIa and bloodborne TF. Despite more than 100,000 simulations, no acceptable fit was obtained by optimization of initial conditions alone. Importantly, adjustment of XIa *(t = 0)* was insufficient to maintain an “engine idling” model of blood consistent with the results in **Figures 9** **–** [Fig pcbi-1000950-g010] **11**. It was necessary to include a source term that generated XIIa at a rate slow enough to have little effect on short time scales in the presence of high concentrations of active proteases, but fast enough to limit *T_i_* to that of the control. TF independent VIIa activity was found to be appreciable at high concentrations and upon active platelet surfaces and was therefore also considered as a proximal trigger of coagulation initiation.

The estimation of a rate of XIIa production (**Reaction 29,** **Table 1**) allows us to extend the predictive capacity of the Hockin-Mann model to low concentrations of TF. Further we were also able to simulate titrations of Va, VIIa, IXa, Xa and XIa in 5× diluted blood **(** **Figure 9** **)**; clotting times observed with additions of TF, IXa, Xa and thrombin in whole blood **(** **Figure 10** **)**; and the observed consequences of platelet activation **(** **Figure 11** **)**. However, the Platelet-Plasma model remains a coarse grained platelet and pseudo-homogeneous model. No distinction is made explicitly between bulk and surface phases and binding of reactants onto the platelet surface is not considered separately. Net conversion rates were sped up as the platelet activates by decreasing the unbinding of the enzyme-substrate complex (representing the role of anionic lipid exposure), but no explicit attempt was made to model the change in exposed surface area and chemistry, and its effect on reaction rates. For the sake of simplicity, a single functional form (ε) was assumed for the changes in rates as the platelet activates, resulting in a two order change in rate of all desorption reactions on the platelet surface. We have shown recently that it is possible to construct donor specific models of platelet calcium mobilization as a function of combinatorial agonist concentrations at the site of the thrombus [57]. To incorporate donor specific descriptions of platelet function in models of coagulation, the correlation between intracellular calcium and PS exposure must be defined.

The present study indicates the future feasibility of a full description of blood accounting for intracellular platelet metabolism and heterogenous reactions on the dynamic platelet surface. In keeping with the static environment in a well plate and the absence of endothelium, no consideration was made of fluid flow, mass transfer from the bulk onto the platelet surface or thrombomodulin/APC mediated pathways. Our model extends the topology of the Hockin-Mann structure to better simulate *in vitro* clotting initiation of whole blood under diverse initial conditions, however the assumption of a well mixed static system is not appropriate for simulations of thrombosis under flow *in vivo*.

Our high throughput measurement system allows us to rapidly and reproducibly quantify the duration of the initiation phase across numerous conditions from a single blood draw. This has the potential to efficiently identify coagulation pathologies. For instance, an extended duration of this metric for a donor would indicate propensity to bleeding diseases like hemophilia while a shortened metric would indicate predisposition to thrombosis. Titration experiments with added proteases provide a higher dimensional examination of blood function than simply measuring the clotting time after addition of high doses of TF.

## Materials and Methods

### Ethics statement

Human blood was collected from consenting normal donors in accordance with the policies of and with the permission of the University of Pennsylvania Institutional Review Board.

### Materials

The fluorogenic thrombin substrates Boc-Val-Pro-Arg-methylcoumarinamide (Boc-VPR-MCA) and Z-Gly-Gly-Arg methylcoumarinamide (Z-GGR-MCA) were obtained from Bachem (King of Prussia, PA). Lipidated recombinant TF (baculovirus expressed, amino acids 1–263; 43kDa), monoclonal anti-human FVII/VIIa, and TF ELISA kits were obtained from American Diagnostica (Stamford, CT) and used to determine the available TF antigen concentration of 18.2 nM. Monoclonal anti human FXI and FXII were obtained from Enzyme Research Laboratories (South bend, IN). Monoclonal anti human P-selectin was obtained from R&D Systems (Minneapolis, MN). Monoclonal anti human Gp1b_α_ was obtained from Lifespan Biosciences (Seattle, WA). Polyclonal anti human cathepsin-G was obtained from Molecular Innovations (Novi, MI). Monoclonal anti human PDI was obtained from was obtained from Affinity Bioreagants (Rockford, IL). Thrombin-antithrombin ELISA kits were obtained from Dade Behring (Deerfield, Illinois). Active site inhibited VIIa (VIIai) was gifted by Novo-Nordisk, Denmark. Other reagents included: adenosine diphosphate (ADP), benzamidine hydrochloride, EDTA, HEPES, NaCl, NaOH, sodium citrate, apyrase, PGE2, Tg003 and Puromycin (all from Sigma, St. Louis, MO); corn trypsin inhibitor (CTI), Phe-Pro-Arg-chloromethylketone (FPRCK), human α-thrombin, polyclonal anti-human TF, Xa, Va, VIIa, IXa, XIa, human plasmas deficient in prothrombin and factors V, VII, XI or XII (all from Hematological Technologies, Essex Junction, VT); convulxin (CVX, Centerchem, Norwalk, CT); and black 384-well plates (Corning, Corning, NY). All reagents were stored and prepared according to the manufacturers' recommendations. The buffer used for all dilutions was HEPES buffered saline (HBS, sterile filtered 20 mM HEPES and 140 mM NaCl in deionized water adjusted to pH 7.4 with NaOH).

### Blood collection and preparation of washed platelets

Unless otherwise noted, blood was collected in a syringe containing 1 part sodium citrate to 9 parts blood and 50 µg CTI/mL. To obtain washed platelets for the experiments in **Figure 8**, blood was first centrifuged at 120g for 12 min to obtain platelet rich plasma (PRP). PRP was then treated with 200µM PGE2 and 1unit/ml apyrase to prevent activation during subsequent spinning. A platelet pellet was obtained by centrifuging PRP at 800g for 10 min and this pellet was resuspended in HBS to yield a final concentration of ∼3×10^8^ platelets/ml.

### High throughput experimentation

Prior to phlebotomy, all reagents were prepared in a 384-well plate using a Thermo Multidrop and a Perkin-Elmer JANUS workstation. For all experiments, blood was added to the whole (or segments) of a well plate using a PerkinElmer Evolution P^3^. To ensure a simultaneous activation time, citrated blood was recalcified in all 384 wells simultaneously, yielding a final added calcium concentration of 10 mM. To detect thrombin activity with time, we added the fluorogenic substrate (S_IIa_), Boc-VPR-MCA (10 µM) to every well and detected the fluorescence of the released aminomethylcoumarin (AMC) with Thermo Fluoroskan fluorimeters preheated to 37°C. Each well was read once per minute for 4 hr. After 4 hours, 5 U thrombin/well was added and then the plate was read again once per minute for 20 minutes to determine the maximal signal. In experiments to study the effect of fluorogenic substrate concentration, Boc-VPR-MCA or Z-GGR-MCA were used at final concentrations between 1 and 100 µM. All experiments were carried out in 5× diluted whole blood. Fractional conversion of the fluorogenic substrate was determined according to:
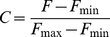
(1)where *C* is the fractional conversion, *F* is the instantaneous fluorescence at any time, *F_min_* is the minimum fluorescence for that well, and *F_max_* is the maximum fluorescence in that well during either the experiment or after thrombin addition. The initiation time (*T_i_*) was set when *C* rises past 0.05.

### Thrombin-Antithrombin (TAT) immunoassays

TAT serves as a measure of the cumulative thrombin production. Citrated blood was dispensed into all wells of a 384-well plate containing calcium using a PerkinElmer Evolution P^3^ liquid handling station. Reactions were manually stopped in columns of the well plate by adding a ‘stop cocktail’ of 50 mM EDTA, 20 mM benzamidine and 100 µM Phe-Pro-Arg-chloromoethyl ketone (PPACK) at specific time points [58]. The plate was spun at 120g for 12 min to separate out plasma, which was subsequently assayed for TAT by ELISA (Dade Behring).

### Simulation

Simulations were performed in Matlab R2008b (version 7.7.0.471) using the Systems Biology Toolbox 2 (SBTOOLBOX2) with the SBPD extension package ODE solver with an absolute tolerance of 10^−30^ and a relative tolerance of 10^−7^ [59].

A function of thrombin concentration was fit with a Hill function to published experimental data that quantified ‘Platelet Activation Status’ by reporting surface Phosphatidylserine exposure (measured by fold increase in Annexin V binding) in response to thrombin (IIa) [60] (**Figure. 2B**):
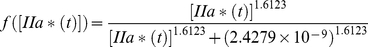
(2)where 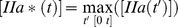. Since thrombin concentration physiologically starts decreasing after monotonically rising to a peak, it was necessary to use the maximum transient thrombin concentration, 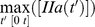, to ensure that 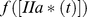 never decreases once it has reached its maximum magnitude. 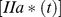 is exactly equivalent to [*IIa(t)*] until [*IIa(t)*] reaches its peak value, whereafter 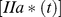 remains constant at that maximum value. This ensures that the platelet stays activated even when thrombin levels decline.

For a given [IIa], we define the maximum platelet activation state, 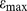:
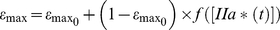
(3)where ε_max_ describes the fractional activation state of the platelet. 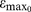 defines the basal activation state of the platelet at *t = 0*, and is set to 0.01 in most simulations (assuming a basal 1% binding strength of coagulation factors to the resting platelet surface). For full activation of platelets at [thrombin] >∼10 nM, 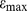 equals 1 and protein dissociation is minimized.

The instantaneous platelet activation state ε is governed by the differential equation
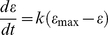
(4)with the initial condition at 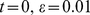, this solves to,
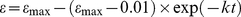
(5)The constant k is inversely proportional to the time scale of platelet activation, and was set to 0.005. This is consistent with the fact that it takes ∼200 s for platelets to mobilize calcium from intracellular stores upon stimulation with thrombin or other platelet agonists [57]. Such a form of the platelet function (ε) ensures that the platelet achieves its maximum attainable activation state (ε_max_) not instantaneously, but on a physiologically relevant timescale. Transients of ε are shown for values of *ε_max_* = 0.25, 0.5, 0.75 and 1.0 are shown in **Figure 2c**.

To account for changes in the reaction rates with platelet activation, we modified the Hockin-Mann model rate constants as follows,
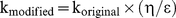
(6)where η was a parameter that was used to alter the magnitude of the rate constant used in the Hockin-Mann model. Many of the Hockin-Mann model parameters were originally fitted empirically to global experimental data. For η = 1 and full platelet activation (ε = 1), the modified value becomes the original value. For η ≠ 1, the modifications may be regarded as further fits to experimental data, consistent with published values of rate constants **(** ***footnotes to*** **Table I)**.

To estimate the sensitivity of our model's output to the choice of kinetic constants, a global sensitivity analysis of the models output thrombin concentration at 0 and 10 pM TF concentration was carried out by the method of weighted averaging of local sensitivities. The results of this analysis are shown in **Figure S1A and Figure S1B in Text S1**, and **Table S1 and Table S2 in Text S1**. Local sensitivity of *T_i_* to 10 fold variations in important parameters (determined from the global sensitivity analysis) across the entire range of titrated TF concentrations is shown in **Figure S1 C in Text S1**.

## Supporting Information

Text S1 A complete description of the ODEs for all 76 species in the assembled reaction network, along with the definition of every reaction rate. Essentially all of the model parameters (rate constants and initial conditions) are known or estimated from literature **(** **Table 1** **)**. The use of η clarifies changes from the original Hockin-Mann rate constants that are justified by more recent literature measurements (See **Table 1** footnotes). Only, the rate of XIIa leakage was estimated (i.e. fitted) based on the difference between simulation and experiment at 0 added T.
(0.54 MB DOC)
