# Supplementary material for: Systems Biology of Coagulation Initiation: Kinetics of Thrombin Generation in Resting and Activated Human Blood
Source: PLoS Comput Biol. 2010 Sep 30;6(9):e1000950. doi: 10.1371/journal.pcbi.1000950 (PMC2947981; doi:10.1371/journal.pcbi.1000950)
Supplement: Text S1 — A complete description of the ODEs for all 76 species in the assembled reaction network, along with the definition of every reaction rate. Essentially all of the model parameters (rate constants and initial conditions) are known or estimated from literature ( Table 1 ). The use of η clarifies changes from the original Hockin-Mann rate constants that are justified by more recent literature measurements (See Table 1 footnotes). Only, the rate of XIIa leakage was estimated (i.e. fitted) based on the difference between simulation and experiment at 0 added T. (0.54 MB DOC) [file pcbi.1000950.s001.doc]

Supporting Information

**Systems biology of coagulation initiation:**

**Kinetics of thrombin generation in resting and activated human blood.**

Manash S. Chatterjee, William S. Denney, Huiyan Jing and Scott L. Diamond^*^

Institute for Medicine and Engineering

Department of Chemical and Biomolecular Engineering

University of Pennsylvania

Philadelphia, PA 19104

USA

^*^Corresponding Author

Scott L. Diamond

1020 Vagelos Research Laboratories

3340 Smith Walk

Philadelphia, PA 19104

[sld@seas.upenn.edu](mailto:sld@seas.upenn.edu)

(215) 573-5702 (Phone)

(215) 573-7227 (Fax)

**Text S1**

**Model Details:**

Rate equations for all of the species in the Platelet-Plasma model

1. d/dt (TF) = Reaction_TF_VII_unbinding - Reaction_TF_VII_binding + Reaction_TF_VIIa_unbinding - Reaction_TF_VIIa_binding
2. d/dt (VII) = Reaction_TF_VII_unbinding - Reaction_TF_VII_binding - Reaction_VII_Activation_by_TF_VIIa - Reaction_VII_Activation_by_Xa -Reaction_VII_Activation_by_IIa
3. d/dt (TF_VII) = -Reaction_TF_VII_unbinding + Reaction_TF_VII_binding
4. d/dt (VIIa) = Reaction_TF_VIIa_unbinding -Reaction_TF_VIIa_binding + Reaction_VII_Activation_by_TF_VIIa + Reaction_VII_Activation_by_Xa + Reaction_VII_Activation_by_IIa - Reaction_X_binding_VIIa + Reaction_X_unbinding_VIIa + Reaction_Xa_production_by_VIIa_alone - Reaction_IX_binding_VIIa + Reaction_IX_unbinding_VIIa + Reaction_IXa_production_by_VIIa_alone
5. d/dt (TF_VIIa) = -Reaction_TF_VIIa_unbinding + Reaction_TF_VIIa_binding + Reaction_X_unbinding_TF_VIIa -Reaction_X_binding_TF_VIIa + Reaction_Xa_unbinding_TF_VIIa - Reaction_Xa_binding_TF_VIIa + Reaction_IX_unbinding_TF_VIIa - Reaction_IX_binding_TF_VIIa + Reaction_IX_activation_by_TF_VIIa - Reaction_Xa_TFPI_binding_TF_VIIa -Reaction_TF_VIIa_binding_ATIII
6. d/dt (Xa) = Reaction_Xa_unbinding_TF_VIIa - Reaction_Xa_binding_TF_VIIa + Reaction_X_activation_by_IXa_VIIIa + Reaction_Xa_unbinding_Va - Reaction_Xa_binding_Va + Reaction_Xa_unbinding_TFPI - Reaction_Xa_binding_TFPI - Reaction_Xa_binding_ATIII + Reaction_Xa_production_by_VIIa_alone + Reaction_Xa_production_by_IXa_alone - Reaction_VIII_binding_Xa +Reaction_VIII_unbinding_Xa +Reaction_VIIIa_production_by_Xa_alone
7. d/dt (IIa) = Reaction_Fbn2_IIa_dissociation - Reaction_Fbn2_IIa_association + Reaction_Fbn2_2_dimer_production + Reaction_Fbn1_2_dimer_unbinding_IIa -Reaction_Fbn1_2_dimer_binding_IIa + Reaction_IIa_producing_Fbn2 + Reaction_Fbn1_unbinding_Fbn1_IIa -Reaction_Fbn1_binding_IIa + Reaction_IIa_producing_Fbn1 + Reaction_Fbg_unbinding_Fbg_IIa - Reaction_Fbg_binding_IIa -Reaction_Boc_VPR_AMC_IIa_binding + Reaction_Boc_VPR_AMC_IIa_unbinding + Reaction_AMC_generation + Reaction_II_activation_by_Xa + Reaction_mIIa_activation_by_Xa_Va -Reaction_IIa_binding_ATIII - Reaction_IIa_binding_XI + Reaction_IIa_unbinding_XI + Reaction_XIa_generation
8. d/dt (X) = Reaction_X_unbinding_TF_VIIa-Reaction_X_binding_TF_VIIa +Reaction_X_unbinding_IXa_VIIIa -Reaction_X_binding_IXa_VIIIa +Reaction_VIIIa_chain_unbinding_IXa_X -Reaction_X_binding_VIIa +Reaction_X_unbinding_VIIa - Reaction_X_binding_IXa +Reaction_X_unbinding_IXa
9. d/dt (TF_VIIa_X) = - Reaction_X_unbinding_TF_VIIa + Reaction_X_binding_TF_VIIa - Reaction_X_activation_by_TF_VIIa
10. d/dt (TF_VIIa_Xa) = + Reaction_X_activation_by_TF_VIIa - Reaction_Xa_unbinding_TF_VIIa + Reaction_Xa_binding_TF_VIIa + Reaction_TFPI_unbinding_TF_VIIa_Xa - Reaction_TFPI_binding_TF_VIIa_Xa
11. d/dt (IX) = +Reaction_IX_unbinding_TF_VIIa-Reaction_IX_binding_TF_VIIa-Reaction_XIa_binding_IX+Reaction_XIa_unbinding_IX-Reaction_IX_binding_VIIa +Reaction_IX_unbinding_VIIa
12. d/dt (TF_VIIa_IX) = -Reaction_IX_unbinding_TF_VIIa + Reaction_IX_binding_TF_VIIa - Reaction_IX_activation_by_TF_VIIa
13. d/dt (IXa) = +Reaction_IX_activation_by_TF_VIIa + Reaction_IXa_unbinding_VIIIa -Reaction_IXa_binding_VIIIa + Reaction_VIIIa_chain_unbinding_IXa_X+Reaction_VIIIa_chain_unbinding_IXa-Reaction_IXa_binding_ATIII + Reaction_XIa_generating_IXa + Reaction_IXa_production_by_VIIa_alone -Reaction_X_binding_IXa + Reaction_X_unbinding_IXa + Reaction_Xa_production_by_IXa_alone
14. d/dt (II) = -Reaction_II_activation_by_Xa+Reaction_II_unbinding_Xa_Va-Reaction_II_binding_Xa_Va
15. d/dt (VIII) = -Reaction_VIII_Activation_by_IIa-Reaction_VIII_binding_Xa + Reaction_VIII_unbinding_Xa
16. d/dt (VIIIa) = +Reaction_VIII_Activation_by_IIa + Reaction_IXa_unbinding_VIIIa - Reaction_IXa_binding_VIIIa + Reaction_VIIIa_chain_rebinding - Reaction_VIIIa_chain_unbinding + Reaction_VIIIa_production_by_Xa_alone
17. d/dt (IXa_VIIIa) = -Reaction_IXa_unbinding_VIIIa + Reaction_IXa_binding_VIIIa + Reaction_X_unbinding_IXa_VIIIa -Reaction_X_binding_IXa_VIIIa + Reaction_X_activation_by_IXa_VIIIa -Reaction_VIIIa_chain_unbinding_IXa
18. d/dt (IXa_VIIIa_X) = - Reaction_X_unbinding_IXa_VIIIa + Reaction_X_binding_IXa_VIIIa - Reaction_X_activation_by_IXa_VIIIa -Reaction_VIIIa_chain_unbinding_IXa_X
19. d/dt (VIIIa1_L) = - Reaction_VIIIa_chain_rebinding + Reaction_VIIIa_chain_unbinding + Reaction_VIIIa_chain_unbinding_IXa_X + Reaction_VIIIa_chain_unbinding_IXa
20. d/dt (VIIIa2) = -Reaction_VIIIa_chain_rebinding + Reaction_VIIIa_chain_unbinding + Reaction_VIIIa_chain_unbinding_IXa_X + Reaction_VIIIa_chain_unbinding_IXa
21. d/dt (V) = -Reaction_V_activation_by_IIa
22. d/dt (Va) = + Reaction_V_activation_by_IIa + Reaction_Xa_unbinding_Va -Reaction_Xa_binding_Va
23. d/dt (Xa_Va) = -Reaction_Xa_unbinding_Va + Reaction_Xa_binding_Va + Reaction_II_unbinding_Xa_Va -Reaction_II_binding_Xa_Va + Reaction_mIIa_dissociation_from_Xa_Va
24. d/dt (Xa_Va_II) = -Reaction_II_unbinding_Xa_Va + Reaction_II_binding_Xa_Va - Reaction_mIIa_dissociation_from_Xa_Va
25. d/dt (mIIa) = + Reaction_mIIa_dissociation_from_Xa_Va -Reaction_mIIa_activation_by_Xa_Va - Reaction_mIIa_binding_ATIII
26. d/dt (TFPI) = + Reaction_Xa_unbinding_TFPI -Reaction_Xa_binding_TFPI + Reaction_TFPI_unbinding_TF_VIIa_Xa - Reaction_TFPI_binding_TF_VIIa_Xa
27. d/dt (Xa_TFPI) = - Reaction_Xa_unbinding_TFPI + Reaction_Xa_binding_TFPI -Reaction_Xa_TFPI_binding_TF_VIIa
28. d/dt (TF_VIIa_Xa_TFPI) = - Reaction_TFPI_unbinding_TF_VIIa_Xa + Reaction_TFPI_binding_TF_VIIa_Xa + Reaction_Xa_TFPI_binding_TF_VIIa
29. d/dt (ATIII) = - Reaction_Fbn2_IIa_destruction_by_ATIII -Reaction_Fbn1_2_IIa_destruction_by_ATIII - Reaction_Fbn1_IIa_destruction_by_ATIII - Reaction_XIIa_destruction_by_ATIII - Reaction_Xa_binding_ATIII - Reaction_mIIa_binding_ATIII - Reaction_IXa_binding_ATIII - Reaction_IIa_binding_ATIII - Reaction_TF_VIIa_binding_ATIII - Reaction_XIa_destruction_by_ATIII
30. d/dt (Xa_ATIII) = + Reaction_Xa_binding_ATIII
31. d/dt (mIIa_ATIII) = + Reaction_mIIa_binding_ATIII
32. d/dt (IXa_ATIII) = + Reaction_IXa_binding_ATIII
33. d/dt (IIa_ATIII) = + Reaction_IIa_binding_ATIII
34. d/dt (TF_VIIa_ATIII) = + Reaction_TF_VIIa_binding_ATIII
35. d/dt (Boc_VPR_AMC) = - Reaction_Boc_VPR_AMC_IIa_binding + Reaction_Boc_VPR_AMC_IIa_unbinding
36. d/dt (Boc_VPR_AMC_IIa) = + Reaction_Boc_VPR_AMC_IIa_binding - Reaction_Boc_VPR_AMC_IIa_unbinding - Reaction_AMC_generation
37. d/dt (Boc_VPR) = + Reaction_AMC_generation
38. d/dt (AMC) = + Reaction_AMC_generation
39. d/dt (XII) = + Reaction_XII_unbinding_Kallikrein_XII -Reaction_XII_binding_Kallikrein - Reaction_XIIa_from_wall_and_platelet -Reaction_XIIa_binding_XII + Reaction_XIIa_unbinding_XII
40. d/dt (XIIa) = + Reaction_Kallikrein_producing_XIIa + Reaction_XIIa_producing_Kallikrein + Reaction_bXIIa_unbinding_PreKallikrein_XIIa -Reaction_bXIIa_binding_PreKallikrein - Reaction_XIIa_destruction_by_ATIII + Reaction_XIIa_from_wall_and_platelet - Reaction_XIIa_binding_XI + Reaction_XIIa_unbinding_XI + Reaction_XIa_production_by_XIIa -Reaction_XIIa_binding_XII + Reaction_XIIa_unbinding_XII + Reaction_autocatalytic_XIIa_amplification - Reaction_XIIa_bindingCTI + Reaction_XIIa_unbindingCTI - Reaction_XIIa_destruction_by_C1inhibitor
41. d/dt (XII_XIIa) = + Reaction_XIIa_binding_XII - Reaction_XIIa_unbinding_XII -Reaction_autocatalytic_XIIa_amplification
42. d/dt (PreKallikrein) = +Reaction_bXIIa_unbinding_PreKallikrein_XIIa -Reaction_bXIIa_binding_PreKallikrein -Reaction_Kallikrein_autoactivation
43. d/dt (PreKallikrein_XIIa) = - Reaction_XIIa_producing_Kallikrein -Reaction_bXIIa_unbinding_PreKallikrein_XIIa + Reaction_bXIIa_binding_PreKallikrein
44. d/dt (Kallikrein_XII) = -Reaction_Kallikrein_producing_XIIa -Reaction_XII_unbinding_Kallikrein_XII + Reaction_XII_binding_Kallikrein
45. d/dt (Kallikrein) = + Reaction_Kallikrein_producing_XIIa + Reaction_XII_unbinding_Kallikrein_XII - Reaction_XII_binding_Kallikrein + Reaction_XIIa_producing_Kallikrein + Reaction_Kallikrein_autoactivation -Reaction_Kallikrein_destruction
46. d/dt (CTI) = - Reaction_XIIa_bindingCTI + Reaction_XIIa_unbindingCTI
47. d/dt (CTI_XIIa) = + Reaction_XIIa_bindingCTI -Reaction_XIIa_unbindingCTI
48. d/dt (C1inhibitor) = -Reaction_XIa_destruction_by_C1inhibitor -Reaction_XIIa_destruction_by_C1inhibitor
49. d/dt (XIIa_C1inhibitor) = + Reaction_XIIa_destruction_by_C1inhibitor
50. d/dt (XIIa_ATIII) = + Reaction_XIIa_destruction_by_ATIII
51. d/dt (XI) = - Reaction_XI_autoactivation - Reaction_IIa_binding_XI + Reaction_IIa_unbinding_XI - Reaction_XIIa_binding_XI + Reaction_XIIa_unbinding_XI
52. d/dt (XI_IIa) = + Reaction_IIa_binding_XI - Reaction_IIa_unbinding_XI -Reaction_XIa_generation
53. d/dt (XIa) = - Reaction_XIa_inhibition_by_alpha2AP -Reaction_XIa_inhibition_by_aplpha1AT + Reaction_XI_autoactivation -Reaction_XIa_binding_IX + Reaction_XIa_unbinding_IX + Reaction_XIa_generating_IXa + Reaction_XIa_generation -Reaction_XIa_destruction_by_ATIII - Reaction_XIa_destruction_by_C1inhibitor + Reaction_XIa_production_by_XIIa
54. d/dt (XIIa_XI) = + Reaction_XIIa_binding_XI - Reaction_XIIa_unbinding_XI -Reaction_XIa_production_by_XIIa
55. d/dt (XIa_ATIII) = + Reaction_XIa_destruction_by_ATIII
56. d/dt (XIa_C1inhibitor) = + Reaction_XIa_destruction_by_C1inhibitor
57. d/dt (alpha1AT) = - Reaction_XIa_inhibition_by_alpha1AT
58. d/dt (alpha2AP) = - Reaction_XIa_inhibition_by_alpha2AP
59. d/dt (XIa_alpha1AT) = + Reaction_XIa_inhibition_by_alpha1AT
60. d/dt (XIa_alpha2AP) = + Reaction_XIa_inhibition_by_alpha2AP
61. d/dt (XIa_IX) = + Reaction_XIa_binding_IX - Reaction_XIa_unbinding_IX-Reaction_XIa_generating_IXa
62. d/dt (IXa_X) = + Reaction_X_binding_IXa - Reaction_X_unbinding_IXa -Reaction_Xa_production_by_IXa_alone
63. d/dt (Xa_VIII) = + Reaction_VIII_binding_Xa - Reaction_VIII_unbinding_Xa - Reaction_VIIIa_production_by_Xa_alone
64. d/dt (IX_VIIa) = + Reaction_IX_binding_VIIa - Reaction_IX_unbinding_VIIa - Reaction_IXa_production_by_VIIa_alone
65. d/dt (X_VIIa) = + Reaction_X_binding_VIIa - Reaction_X_unbinding_VIIa - Reaction_Xa_production_by_VIIa_alone
66. d/dt (Fbg) = + Reaction_Fbg_unbinding_Fbg_IIa -Reaction_Fbg_binding_IIa
67. d/dt (Fbg_IIa) = -Reaction_IIa_producing_Fbn1 -Reaction_Fbg_unbinding_Fbg_IIa + Reaction_Fbg_binding_IIa
68. d/dt (Fbn1) = + Reaction_Fbn1_2_dimer_dissociation -Reaction_Fbn1_2_dimer_association + Reaction_Fbn1_unbinding_Fbn1_IIa -Reaction_Fbn1_binding_IIa + Reaction_IIa_producing_Fbn1
69. d/dt (Fbn1_IIa) = - Reaction_Fbn1_IIa_destruction_by_ATIII -Reaction_IIa_producing_Fbn2 - Reaction_Fbn1_unbinding_Fbn1_IIa + Reaction_Fbn1_binding_IIa
70. d/dt (Fbn1_2) = + Reaction_Fbn1_2_dimer_unbinding_IIa - Reaction_Fbn1_2_dimer_binding_IIa - Reaction_Fbn1_2_dimer_dissociation + Reaction_Fbn1_2_dimer_association
71. d/dt (Fbn1_2_IIa) = - Reaction_Fbn2_2_dimer_production -Reaction_Fbn1_2_dimer_unbinding_IIa + Reaction_Fbn1_2_dimer_binding_IIa -Reaction_Fbn1_2_IIa_destruction_by_ATIII
72. d/dt (Fbn2) = + Reaction_Fbn2_IIa_dissociation -Reaction_Fbn2_IIa_association + Reaction_IIa_producing_Fbn2
73. d/dt (Fbn2_IIa) = - Reaction_Fbn2_IIa_destruction_by_ATIII -Reaction_Fbn2_IIa_dissociation + Reaction_Fbn2_IIa_association
74. d/dt (Fbn1_2_IIa_ATIII) = + Reaction_Fbn1_2_IIa_destruction_by_ATIII
75. d/dt (Fbn1_IIa_ATIII) = + Reaction_Fbn1_IIa_destruction_by_ATIII
76. d/dt (Fbn2_IIa_ATIII) = + Reaction_Fbn2_IIa_destruction_by_ATIII

Model Reactions

1. Reaction_TF_VII_unbinding = k_off_TF_VII × [TF_VII]
2. Reaction_TF_VII_binding = k_on_TF_VII × [TF] × [VII]
3. Reaction_TF_VIIa_unbinding = k_off_TF_VIIa × [TF_VIIa]
4. Reaction_TF_VIIa_binding = k_on_TF_VIIa× [TF] × [VIIa]
5. Reaction_VII_Activation_by_TF_VIIa = k_cat_VII_TF_VIIa × [VII]× [TF_VIIa]
6. Reaction_VII_Activation_by_Xa = k_cat_VII_Xa × [VII]× [Xa]
7. Reaction_VII_Activation_by_IIa = k_cat_VII_IIa × [VII] × [IIa]
8. Reaction_X_binding_VIIa = _k_on_X_VIIa × [VIIa] × [X]
9. Reaction_X_unbinding_VIIa = _k_off_X_VIIa × [X_VIIa] / ε
10. Reaction_Xa_production_by_VIIa_alone = _k_cat_X_VIIa × [X_VIIa]
11. Reaction_IX_binding_VIIa = _k_on_IX_VIIa × [VIIa] × [IX]
12. Reaction_IX_unbinding_VIIa = _k_off_IX_VIIa × [IX_VIIa]
13. Reaction_IXa_production_by_VIIa_alone = _k_cat_IX_VIIa × [IX_VIIa]
14. Reaction_X_unbinding_TF_VIIa = k_off_X_TF_VIIa × [TF_VIIa_X]
15. Reaction_X_binding_TF_VIIa = k_on_X_TF_VIIa × [TF_VIIa] × [X]
16. Reaction_Xa_unbinding_TF_VIIa = k_off_Xa_TF_VIIa× [TF_VIIa_Xa]
17. Reaction_Xa_binding_TF_VIIa = k_on_Xa_TF_VIIa × [TF_VIIa] × [Xa]
18. Reaction_IX_unbinding_TF_VIIa = k_off_IX_TF_VIIa × [TF_VIIa_IX]
19. Reaction_IX_binding_TF_VIIa = k_on_IX_TF_VIIa × [TF_VIIa] × [IX]
20. Reaction_IX_activation_by_TF_VIIa = k_cat_IX_TF_VIIa × [TF_VIIa_IX]
21. Reaction_Xa_TFPI_binding_TF_VIIa = k_on_TF_VIIa_Xa_TFPI × [TF_VIIa] × [Xa_TFPI]
22. Reaction_TF_VIIa_binding_ATIII = k_on_TF_VIIa_ATIII × [TF_VIIa] × [ATIII]
23. Reaction_X_activation_by_IXa_VIIIa = k_cat_X_IXa_VIIIa × [IXa_VIIIa_X]
24. Reaction_Xa_unbinding_Va = k_off_Xa_Va × [Xa_Va] / ε
25. Reaction_Xa_binding_Va = k_on_Xa_Va × [Xa] × [Va]
26. Reaction_Xa_unbinding_TFPI = k_off_Xa_TFPI × [Xa_TFPI] / ε
27. Reaction_Xa_binding_TFPI = k_on_Xa_TFPI × [Xa] × [TFPI]
28. Reaction_Xa_binding_ATIII = k_on_Xa_ATIII × [Xa] × [ATIII]
29. Reaction_Xa_production_by_VIIa_alone = _k_cat_X_VIIa × [X_VIIa]
30. Reaction_Xa_production_by_IXa_alone=_k_cat_IXa_X × [IXa_X]
31. Reaction_VIII_binding_Xa=_k_on_Xa_VIII × [Xa] × [VIII]
32. Reaction_VIII_unbinding_Xa=_k_off_Xa_VIII × [Xa_VIII] / ε
33. Reaction_VIIIa_production_by_Xa_alone=_k_cat_Xa_VIII × [Xa_VIII]
34. Reaction_Fbn2_IIa_dissociation = k_off_Fbn2_IIa_dissociation × [Fbn2_IIa]
35. Reaction_Fbn2_IIa_association = k_on_Fbn2_IIa_association × [IIa] × [Fbn2]
36. Reaction_Fbn2_2_dimer_production = k_cat_IIa_producing_Fbn2_2 × [Fbn1_2_IIa]
37. Reaction_Fbn1_2_dimer_unbinding_IIa = k_off_Fbn1_2_unbinding_Fbn1_IIa × [Fbn1_2_IIa]
38. Reaction_Fbn1_2_dimer_binding_IIa = k_on_Fbn1_2_binding_IIa × [IIa] × [Fbn1_2]
39. Reaction_IIa_producing_Fbn2 = k_cat_IIa_producing_Fbn2 × [Fbn1_IIa]
40. Reaction_Fbn1_unbinding_Fbn1_IIa = k_off_Fbn1_unbinding_Fbn1_IIa × [Fbn1_IIa]
41. Reaction_Fbn1_binding_IIa = k_on_Fbn1_binding_IIa × [IIa] × [Fbn1]
42. Reaction_IIa_producing_Fbn1 = k_cat_IIa_producing_Fbn1 × [Fbg_IIa]
43. Reaction_Fbg_unbinding_Fbg_IIa = k_off_Fbg_unbinding_Fbg_IIa × [Fbg_IIa]
44. Reaction_Fbg_binding_IIa = k_on_Fbg_binding_IIa × [IIa] × [Fbg]
45. Reaction_Boc_VPR_AMC_IIa_binding = k_on_Boc_VPR_AMC_IIa × [Boc_VPR_AMC] × [IIa]
46. Reaction_Boc_VPR_AMC_IIa_unbinding = k_off_Boc_VPR_AMC_IIa × [Boc_VPR_AMC_IIa]
47. Reaction_AMC_generation = k_cat_Boc_VPR_AMC_IIa × [Boc_VPR_AMC_IIa]
48. Reaction_II_activation_by_Xa = k_cat_II_Xa × [II] × [Xa]
49. Reaction_mIIa_activation_by_Xa_Va = k_cat_mIIa_Xa_Va × [mIIa] × [Xa_Va]
50. Reaction_IIa_binding_ATIII = k_on_IIa_ATIII × [IIa] × [ATIII]
51. Reaction_IIa_binding_XI = k_on_XI_IIa × [XI] × [IIa]
52. Reaction_IIa_unbinding_XI = k_off_XI_IIa × [XI_IIa]
53. Reaction_XIa_generation = k_cat_XI_IIa × [XI_IIa]
54. Reaction_X_unbinding_IXa_VIIIa = k_off_X_IXa_VIIIa× [IXa_VIIIa_X] / ε
55. Reaction_X_binding_IXa_VIIIa = k_on_X_IXa_VIIIa × [X] × [IXa_VIIIa]
56. Reaction_VIIIa_chain_unbinding_IXa_X = k_off_VIII_HC_LC_IXa_X × [IXa_VIIIa_X]
57. Reaction_X_binding_IXa= k_on_IXa_X × [IXa] × [X]
58. Reaction_X_unbinding_IXa= k_off_IXa_X × [IXa_X] / ε
59. Reaction_X_activation_by_TF_VIIa = k_cat_X_TF_VIIa × [TF_VIIa_X]
60. Reaction_TFPI_unbinding_TF_VIIa_Xa = k_off_TFPI_TF_VIIa_Xa × [TF_VIIa_Xa_TFPI]
61. Reaction_TFPI_binding_TF_VIIa_Xa = k_on_TFPI_TF_VIIa_Xa × [TF_VIIa_Xa] × [TFPI]
62. Reaction_XIa_binding_IX = k_on_XIa_IX × [XIa] × [IX]
63. Reaction_XIa_unbinding_IX = k_off_XIa_IX × [XIa_IX] /ε
64. Reaction_IXa_unbinding_VIIIa = k_off_IXa_VIIIa × [IXa_VIIIa] / ε
65. Reaction_IXa_binding_VIIIa = k_on_IXa_VIIIa × [IXa] × [VIIIa]
66. Reaction_VIIIa_chain_unbinding_IXa_X = k_off_VIII_HC_LC_IXa_X × [IXa_VIIIa_X]
67. Reaction_VIIIa_chain_unbinding_IXa = k_off_VIII_HC_LC_IXa × [IXa_VIIIa]
68. Reaction_IXa_binding_ATIII = k_on_IXa_ATIII × [IXa] × [ATIII]
69. Reaction_XIa_generating_IXa = k_cat_XIa_IX× [XIa_IX]
70. Reaction_II_unbinding_Xa_Va = k_off_II_Xa_Va ×[Xa_Va_II] / ε
71. Reaction_II_binding_Xa_Va = k_on_II_Xa_Va × [II] × [Xa_Va]
72. Reaction_VIII_Activation_by_IIa = k_cat_VIII_IIa × [VIII] × [IIa]
73. Reaction_VIIIa_chain_rebinding = k_on_VIII_HC_LC × [VIIIa1_L] × [VIIIa2]
74. Reaction_VIIIa_chain_unbinding = k_off_VIII_HC_LC × [VIIIa] / ε
75. Reaction_V_activation_by_IIa = k_cat_V_IIa × [V] × [IIa]
76. Reaction_mIIa_dissociation_from_Xa_Va = k_cat_off_mIIa_Xa_Va × [Xa_Va_II]
77. Reaction_mIIa_binding_ATIII = k_on_mIIa_ATIII × [mIIa] × [ATIII]
78. Reaction_Fbn2_IIa_destruction_by_ATIII = k_on_Fbn2_IIa_destruction_by_ATIII × [ATIII] × [Fbn2_IIa]
79. Reaction_Fbn1_2_IIa_destruction_by_ATIII = k_on_Fbn1_2_destruction_by_ATIII × [ATIII] × [Fbn1_2_IIa]
80. Reaction_Fbn1_IIa_destruction_by_ATIII = k_on_Fbn1_IIa_destruction_by_ATIII × [ATIII] × [Fbn1_IIa]
81. Reaction_XIIa_destruction_by_ATIII = k_on_ATIII_inhibition_of_XIIa × [XIIa] × [ATIII]
82. Reaction_XIa_destruction_by_ATIII= k_on_ATIII_inhibition_of_XIa × [XIa] × [ATIII]
83. Reaction_XII_unbinding_Kallikrein_XII = k_off_XII_unbinding_Kallikrein_XII × [Kallikrein_XII] / ε
84. Reaction_XII_binding_Kallikrein = k_on_XII_binding_Kallikrein × [Kallikrein] × [XII]
85. Reaction_XIIa_from_wall_and_platelet = k_on_Wall_XIIa_production_rate × [XII]
86. Reaction_XIIa_binding_XII = k_on_XII_XIIa × [XII] × [XIIa]
87. Reaction_XIIa_unbinding_XII = k_off_XII_XIIa × [XII_XIIa] / ε
88. Reaction_Kallikrein_producing_XIIa = k_cat_Kallikrein_producing_XIIa × [Kallikrein_XII]
89. Reaction_XIIa_producing_Kallikrein = k_cat_XIIa_producing_Kallikrein × [PreKallikrein_XIIa]
90. Reaction_bXIIa_unbinding_PreKallikrein_XIIa = k_off_bXIIa_unbinding_PreKallikrein_XIIa × [PreKallikrein_XIIa] / ε
91. Reaction_bXIIa_binding_PreKallikrein = k_on_bXIIa_binding_PreKallikrein × [PreKallikrein] × [XIIa]
92. Reaction_XIIa_binding_XI = k_on_XIIa_XI × [XIIa] × [XI]
93. Reaction_XIIa_unbinding_XI = k_off_XIIa_XI × [XIIa_XI] / ε
94. Reaction_XIa_production_by_XIIa= k_cat_XIIa_XI × [XIIa_XI]
95. Reaction_autocatalytic_XIIa_amplification = k_cat_XIIa_production × [XII_XIIa]
96. Reaction_XIIa_bindingCTI= k_on_CTI_XIIa × [XIIa] × [CTI]
97. Reaction_XIIa_unbindingCTI= k_off_CTI_XIIa × [CTI_XIIa]
98. Reaction_XIIa_destruction_by_C1inhibitor = k_on_C1inh_inhibition_of_XIIa × [XIIa] × [C1inhibitor]
99. Reaction_Kallikrein_autoactivation = k_on_Kallikrein_autoactivation × [PreKallikrein] × [Kallikrein]
100. Reaction_Kallikrein_destruction= k_on_Kallikrein_inhibition × [Kallikrein]
101. Reaction_XIa_destruction_by_C1inhibitor = k_on_C1inh_inhibition_of_XIa × [XIa] × [C1inhibitor]
102. Reaction_XI_autoactivation= k_on_XI_autoactivation × [XI] × [XIa]
103. Reaction_XIa_inhibition_by_alpha2AP = k_on_XIa_inhibition_by_alpha2AP × [XIa] × [alpha2AP]
104. Reaction_XIa_inhibition_by_alpha1AT = k_on_XIa_inhibition_by_alpha1AT × [XIa] × [alpha1AT]
105. Reaction_Fbn1_2_dimer_dissociation= k_off_Fbn1_2_dimer_dissociation × [Fbn1_2_Iia]
106. Reaction_Fbn1_2_dimer_association = k_on_Fbn1_2_dimer_association × [Fbn1] × [Fbn1]


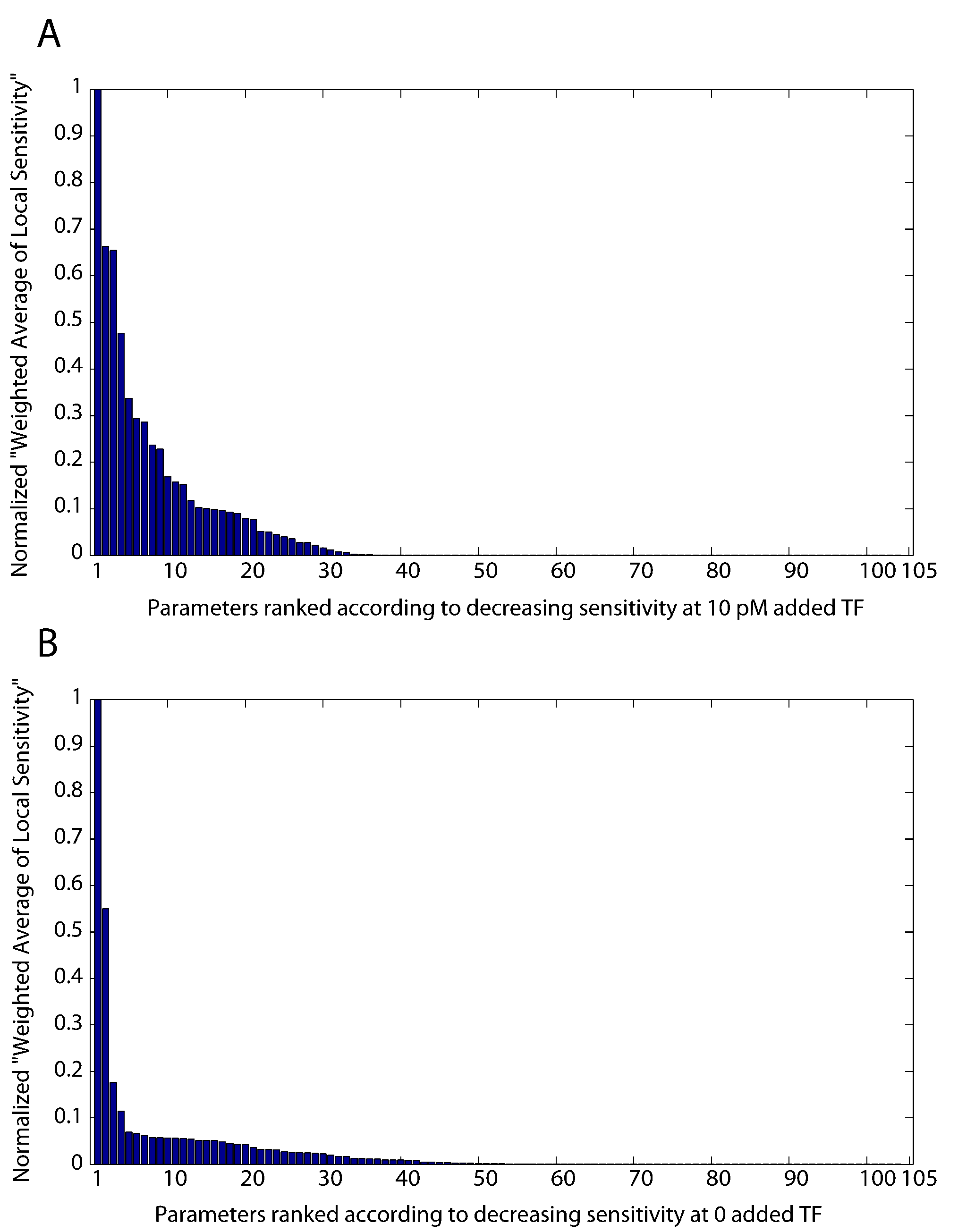


**Figure S1: Global Sensitivity Analysis of the Platelet-Plasma** model

| **Rank** | **Parameter** | **Rank** | **Parameter** |
| --- | --- | --- | --- |
| 1 | 'k_on_TF_VIIa' | 54 | 'k_on_Fbn1_binding_IIa' |
| 2 | 'k_on_X_TF_VIIa' | 55 | 'k_off_X_VIIa' |
| 3 | 'k_off_IX_VIIa' | 56 | 'k_off_IXa_X' |
| 4 | 'k_on_IX_VIIa' | 57 | 'k_on_IXa_X' |
| 5 | 'k_cat_VII_Xa' | 58 | 'k_off_Xa_VIII' |
| 6 | 'k_cat_V_IIa' | 59 | 'k_off_Fbn1_2_unbinding_Fbn1_IIa' |
| 7 | 'k_on_Xa_Va' | 60 | 'k_cat_Xa_VIII' |
| 8 | 'k_off_Xa_Va' | 61 | 'k_on_TF_VIIa_Xa_TFPI' |
| 9 | 'k_cat_II_Xa' | 62 | 'k_cat_VII_IIa' |
| 10 | 'k_cat_mIIa_Xa_Va' | 63 | 'k_on_IXa_ATIII' |
| 11 | 'k_on_II_Xa_Va' | 64 | 'k_off_XIIa_XI' |
| 12 | 'k_off_Xa_TF_VIIa' | 65 | 'k_off_VIII_HC_LC_IXa' |
| 13 | 'k_cat_X_TF_VIIa' | 66 | 'k_cat_XIIa_producing_Kallikrein' |
| 14 | 'k_on_TF_VII' | 67 | 'k_on_bXIIa_binding_PreKallikrein' |
| 15 | 'k_on_TFPI_TF_VIIa_Xa' | 68 | 'k_on_wall_XIIa_production' |
| 16 | 'k_off_TF_VII' | 69 | 'k_off_XII_unbinding_Kallikrein_XII' |
| 17 | 'k_off_XI_IIa' | 70 | 'k_off_XII_XIIa' |
| 18 | 'k_on_XI_IIa' | 71 | 'k_on_Xa_VIII' |
| 19 | 'k_cat_off_mIIa_Xa_Va' | 72 | 'k_on_CTI_XIIa' |
| 20 | 'k_off_TFPI_TF_VIIa_Xa' | 73 | 'k_cat_XI_IIa' |
| 21 | 'k_off_II_Xa_Va' | 74 | 'k_off_X_IXa_VIIIa' |
| 22 | 'k_on_IIa_ATIII' | 75 | 'k_on_Fbn1_2_destruction_by_ATIII' |
| 23 | 'k_on_Xa_ATIII' | 76 | 'k_cat_XIIa_XI' |
| 24 | 'k_on_Fbg_binding_IIa' | 77 | 'k_off_CTI_XIIa' |
| 25 | 'k_off_Fbg_unbinding_Fbg_IIa' | 78 | 'k_on_XI_autoactivation' |
| 26 | 'k_plt_delay' | 79 | 'k_off_VIII_HC_LC_IXa_X' |
| 27 | 'k_off_Boc_VPR_AMC_IIa' | 80 | 'k_off_VIII_HC_LC' |
| 28 | 'k_on_Boc_VPR_AMC_IIa' | 81 | 'k_off_Fbn2_IIa_dissociation' |
| 29 | 'k_on_IX_TF_VIIa' | 82 | 'k_on_Fbn2_IIa_association' |
| 30 | 'k_off_IX_TF_VIIa' | 83 | 'k_on_XII_binding_Kallikrein' |
| 31 | 'k_on_mIIa_ATIII' | 84 | 'k_on_C1inh_inhibition_of_XIIa' |
| 32 | 'k_cat_IIa_producing_Fbn1' | 85 | 'k_on_Fbn2_IIa_destruction_by_ATIII' |
| 33 | 'k_cat_IX_TF_VIIa' | 86 | 'k_on_XIa_IX' |
| 34 | 'k_on_TF_VIIa_ATIII' | 87 | 'k_on_ATIII_inhibition_of_XIIa' |
| 35 | 'k_off_Xa_TFPI' | 88 | 'k_on_XIa_inhibition_by_alpha1AT' |
| 36 | 'k_on_Xa_TFPI' | 89 | 'k_on_Kallikrein_autoactivation' |
| 37 | 'k_on_IXa_VIIIa' | 90 | 'k_on_C1inh_inhibition_of_XIa' |
| 38 | 'k_cat_VIII_IIa' | 91 | 'k_cat_IIa_producing_Fbn2' |
| 39 | 'k_on_X_IXa_VIIIa' | 92 | 'k_on_XIa_inhibition_by_alpha2AP' |
| 40 | 'k_cat_Boc_VPR_AMC_IIa' | 93 | 'k_on_VIII_HC_LC' |
| 41 | 'k_off_Fbn1_unbinding_Fbn1_IIa' | 94 | 'k_cat_X_VIIa' |
| 42 | 'k_off_TF_VIIa' | 95 | 'k_on_XIIa_XI' |
| 43 | 'k_off_X_TF_VIIa' | 96 | 'k_on_XII_XIIa' |
| 44 | 'k_on_Fbn1_2_dimer_association' | 97 | 'k_cat_XIa_IX' |
| 45 | 'k_on_Xa_TF_VIIa' | 98 | 'k_cat_Kallikrein_producing_XIIa' |
| 46 | 'k_off_IXa_VIIIa' | 99 | 'k_cat_IIa_producing_Fbn2_2' |
| 47 | 'k_cat_IXa_X' | 100 | 'k_on_Kallikrein_inhibition' |
| 48 | 'k_cat_X_IXa_VIIIa' | 101 | 'k_cat_XIIa_production' |
| 49 | 'k_on_Fbn1_IIa_destruction_by_ATIII' | 102 | 'k_off_XIa_IX' |
| 50 | 'k_on_X_VIIa' | 103 | 'k_off_bXIIa_unbinding_PreKallikrein_XIIa' |
| 51 | 'k_cat_IX_VIIa' | 104 | 'k_on_ATIII_inhibition_of_XIa' |
| 52 | 'k_on_Fbn1_2_binding_IIa' | 105 | 'k_off_Fbn1_2_dimer_dissociation' |
| 53 | 'k_cat_VII_TF_VIIa' |  |  |

Table S1: Rank Order of Sensitivities at 10 pM added TF

| **Rank** | **Parameter** | **Rank** | **Parameter** |
| --- | --- | --- | --- |
| 1 | 'k_on_XI_autoactivation' | 54 | 'k_off_VIII_HC_LC_IXa' |
| 2 | 'k_on_IIa_ATIII' | 55 | 'k_off_Xa_TFPI' |
| 3 | 'k_on_C1inh_inhibition_of_XIa' | 56 | 'k_on_XII_binding_Kallikrein' |
| 4 | 'k_on_XIa_inhibition_by_alpha1AT' | 57 | 'k_cat_Kallikrein_producing_XIIa' |
| 5 | 'k_on_ATIII_inhibition_of_XIa' | 58 | 'k_cat_XIIa_producing_Kallikrein' |
| 6 | 'k_cat_II_Xa' | 59 | 'k_on_bXIIa_binding_PreKallikrein' |
| 7 | 'k_off_XIa_IX' | 60 | 'k_off_XII_unbinding_Kallikrein_XII' |
| 8 | 'k_on_XIa_IX' | 61 | 'k_on_C1inh_inhibition_of_XIIa' |
| 9 | 'k_cat_XIa_IX' | 62 | 'k_off_bXIIa_unbinding_PreKallikrein_XIIa' |
| 10 | 'k_off_XIIa_XI' | 63 | 'k_on_Kallikrein_inhibition' |
| 11 | 'k_cat_VIII_IIa' | 64 | 'k_on_mIIa_ATIII' |
| 12 | 'k_on_Fbg_binding_IIa' | 65 | 'k_cat_XI_IIa' |
| 13 | 'k_on_CTI_XIIa' | 66 | 'k_on_Xa_VIII' |
| 14 | 'k_on_Fbn2_IIa_destruction_by_ATIII' | 67 | 'k_cat_Xa_VIII' |
| 15 | 'k_cat_XIIa_XI' | 68 | 'k_off_Xa_VIII' |
| 16 | 'k_on_XIIa_XI' | 69 | 'k_on_IXa_ATIII' |
| 17 | 'k_off_CTI_XIIa' | 70 | 'k_off_Fbn1_2_unbinding_Fbn1_IIa' |
| 18 | 'k_off_Fbn2_IIa_dissociation' | 71 | 'k_on_Fbn1_2_binding_IIa' |
| 19 | 'k_on_wall_XIIa_production' | 72 | 'k_on_ATIII_inhibition_of_XIIa' |
| 20 | 'k_off_Fbg_unbinding_Fbg_IIa' | 73 | 'k_off_VIII_HC_LC_IXa_X' |
| 21 | 'k_on_Fbn2_IIa_association' | 74 | 'k_on_Kallikrein_autoactivation' |
| 22 | 'k_on_Fbn1_2_dimer_association' | 75 | 'k_cat_IIa_producing_Fbn2_2' |
| 23 | 'k_off_Fbn1_unbinding_Fbn1_IIa' | 76 | 'k_on_Fbn1_2_destruction_by_ATIII' |
| 24 | 'k_cat_IIa_producing_Fbn1' | 77 | 'k_off_Fbn1_2_dimer_dissociation' |
| 25 | 'k_on_X_IXa_VIIIa' | 78 | 'k_cat_VII_Xa' |
| 26 | 'k_on_IXa_VIIIa' | 79 | 'k_on_VIII_HC_LC' |
| 27 | 'k_cat_IXa_X' | 80 | 'k_cat_X_VIIa' |
| 28 | 'k_off_IXa_X' | 81 | 'k_off_XII_XIIa' |
| 29 | 'k_on_Fbn1_binding_IIa' | 82 | 'k_cat_VII_IIa' |
| 30 | 'k_cat_IIa_producing_Fbn2' | 83 | 'k_on_X_VIIa' |
| 31 | 'k_on_IXa_X' | 84 | 'k_cat_XIIa_production' |
| 32 | 'k_cat_V_IIa' | 85 | 'k_on_XII_XIIa' |
| 33 | 'k_cat_X_IXa_VIIIa' | 86 | 'k_off_X_VIIa' |
| 34 | 'k_on_XIa_inhibition_by_alpha2AP' | 87 | 'k_off_IX_TF_VIIa' |
| 35 | 'k_on_Boc_VPR_AMC_IIa' | 88 | 'k_on_TF_VII' |
| 36 | 'k_on_Xa_ATIII' | 89 | 'k_off_TF_VII' |
| 37 | 'k_off_Xa_Va' | 90 | 'k_on_TFPI_TF_VIIa_Xa' |
| 38 | 'k_off_II_Xa_Va' | 91 | 'k_on_TF_VIIa_Xa_TFPI' |
| 39 | 'k_on_II_Xa_Va' | 92 | 'k_on_Xa_TF_VIIa' |
| 40 | 'k_off_VIII_HC_LC' | 93 | 'k_off_TF_VIIa' |
| 41 | 'k_on_Xa_Va' | 94 | 'k_cat_IX_TF_VIIa' |
| 42 | 'k_on_XI_IIa' | 95 | 'k_off_Xa_TF_VIIa' |
| 43 | 'k_cat_mIIa_Xa_Va' | 96 | 'k_on_TF_VIIa_ATIII' |
| 44 | 'k_cat_off_mIIa_Xa_Va' | 97 | 'k_off_TFPI_TF_VIIa_Xa' |
| 45 | 'k_off_IXa_VIIIa' | 98 | 'k_on_IX_TF_VIIa' |
| 46 | 'k_cat_IX_VIIa' | 99 | 'k_on_TF_VIIa' |
| 47 | 'k_off_Boc_VPR_AMC_IIa' | 100 | 'k_off_X_IXa_VIIIa' |
| 48 | 'k_plt_delay' | 101 | 'k_on_Fbn1_IIa_destruction_by_ATIII' |
| 49 | 'k_off_XI_IIa' | 102 | 'k_off_X_TF_VIIa' |
| 50 | 'k_cat_Boc_VPR_AMC_IIa' | 103 | 'k_cat_X_TF_VIIa' |
| 51 | 'k_off_IX_VIIa' | 104 | 'k_on_X_TF_VIIa' |
| 52 | 'k_on_IX_VIIa' | 105 | 'k_cat_VII_TF_VIIa' |
| 53 | 'k_on_Xa_TFPI' |  |  |

**Table S2: Rank Order of Sensitivities at 0 added TF**

**Global Sensitivity Analysis of the Platelet-Plasma Model**

We estimated global parameter sensitivities for the Platelet-Plasma Model’s output for 10 pM added TF **(Figure S1A and Table S1)** and 0 added TF **(Figure S1B and Table S2)** input stimuli by the method by the method of weighted averaging of local sensitivities [1].

In this method local parameter sensitivities in output ‘*y’*, for a parameter ‘*j’* (j = 1, …, 105) at a point *‘i’* in parameter space is first calculated by introducing a 2% increment in the parameter *p_j_* :

$s_{ij}=\frac{\partial y/y}{\partial p_{j}/p_{j}}$ (1)

We define ‘*y’* to be the thrombin concentration at the initiation time (*T_i_*) of the nominal parameter set. Note *T_i_* cannot be used as the output during sensitivity analysis because 5% conversion of the fluorogenic substrate is not achieved in some perturbed parameter sets.

Local sensitivities were calculated at multiple random points *‘i’* in a very large parameter space where each of the 105 parameters has a spread of half an order of magnitude around their nominal value. In total 10,000 points were explored (10,000 ×105 simulations) and global sensitivity indices were determined by calculating a weighted average of local sensitivities. Similar to Bentele *et al*. [1] we use the Boltzmann- Distribution exp (-E / k_b_T) for weighting, in which E is the difference in thrombin concentration between the nominal and perturbed states and k_b_T is a scaling factor (we use minimum E for scaling). Based on the assumption that parameter sets resulting in output values close to the experimental observation are the most probable, this approach statistically amplifies the sensitivities of parameter sets that result in outputs close to the nominal solution. For ease of visualization sensitivity indices were normalized between 0 and 1 and the values for ranked according o decreasing sensitivity.

Other methods of global sensitivity analysis like ‘Sobol’s method’ [2] or the ‘Extended FAST method’ [3] provide non-biased sampling over the entire parameter space . Such methods were attempted *(not shown)* and provide similar rank orderings of sensitivities for the most sensitive parameters. However convergence was not achieved in a feasible amount of time.

For 10 pM added TF **(Figure S1A and Table S1),** the rate of binding of TF to VIIa was found to be the most sensitive, followed by the rate of binding of X to TF:VIIa. Interestingly, thrombin production was also found to be strongly sensitive to TF independent VIIa binding to IX, as well as VIIa ‘s ability to convert X by itself. Reactions regulating prothrombinase formation (Ranks 7 and 8) were also found to be sensitive. Contact activation (XIIa) mediated reaction rates were generally too slow too have any appreciable effect, although thrombin feedback on XI was found to be moderately sensitive (Ranks 17 and 18)

In the absence of external TF **(Figure S1B and Table S2)**, regulation of XIa activity resulting from its production by autoactivation or inhibition by C1-inhibitor, alpha1- antitrypsin or ATIII its was found to be the most sensitive. The estimated XIIa production rate was moderately sensitive (Rank 19) but not the most crucial determinant of contact activation in the presence of CTI. This is in accordance with our observation that very minute amounts of XIIa, leakage past CTI can strongly self amplify via XIa. On the slow timescales of activation in these simulations, inhibition of thrombin activity by ATIII or by its binding to fibrinogen was also found to be important. On expected lines, no sensitivity to TF mediated reactions was observed.


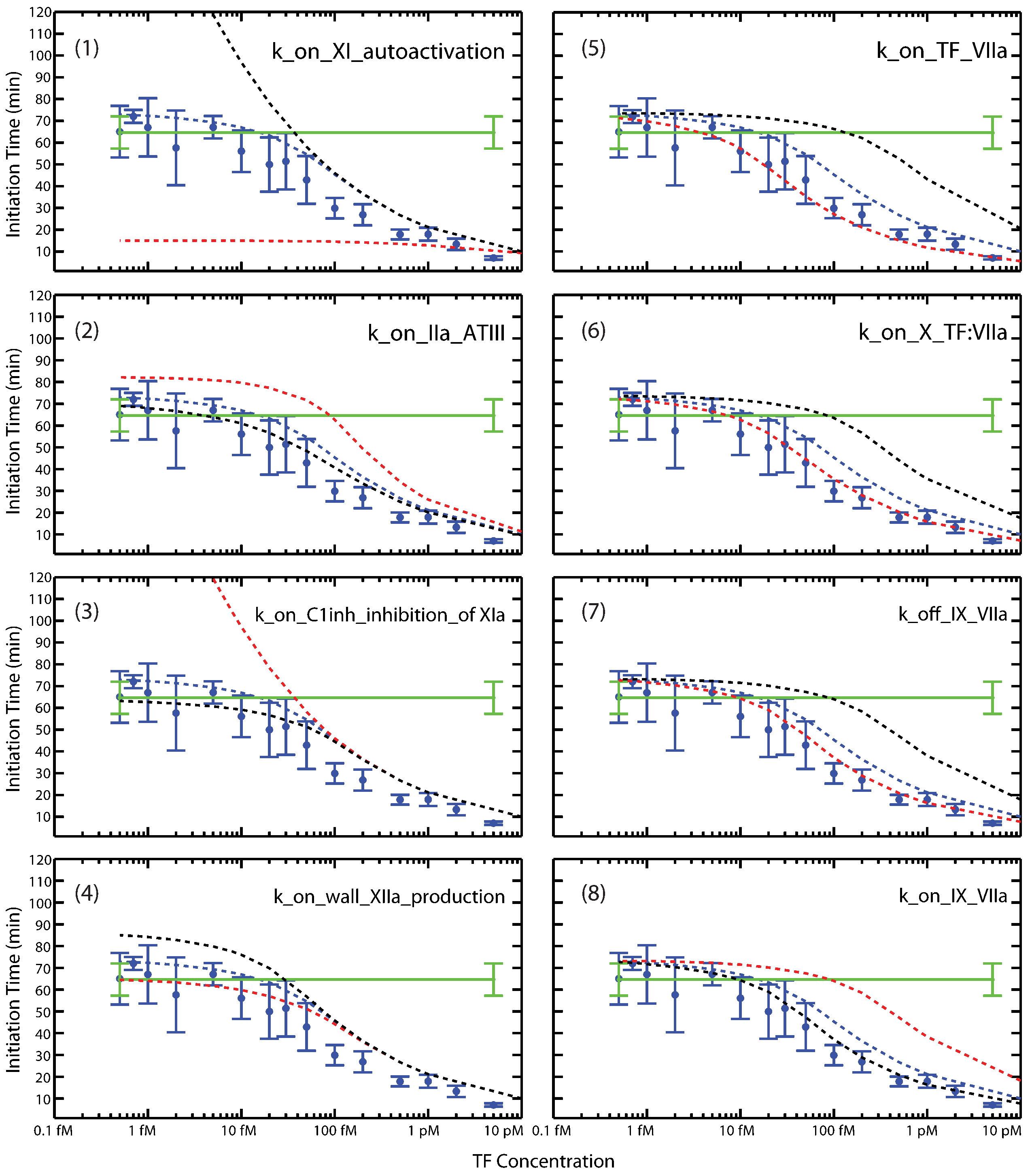


**Figure S1 C: Effect of variation of important parameters of the Platelet-Plasma model across a TF titration.**

The experimental values of T_i_ for a titration of TF are shown in *blue*. The experimental value of the control is shown in *green*. Simulated values of T_i_ are shown by the *blue dashed line.* Important individual parameters were locally perturbed by either 10× *(red dashed lined)* or 0.10× *(black dashed line)* and the resulting simulated titrations are shown above. This local sensitivity analysis was performed for the 3 most globally sensitive parameters *(subplots 1-3)* in the absence of added TF **(Figure S1B and Table S2)**, the estimated rate of XIIa produced from the wall *(subplot 4)*, as well as for the 4 most globally sensitive parameters *(subplots 5-8)* in the presence of 10pM added TF **(Figure S1A and Table S1)**.


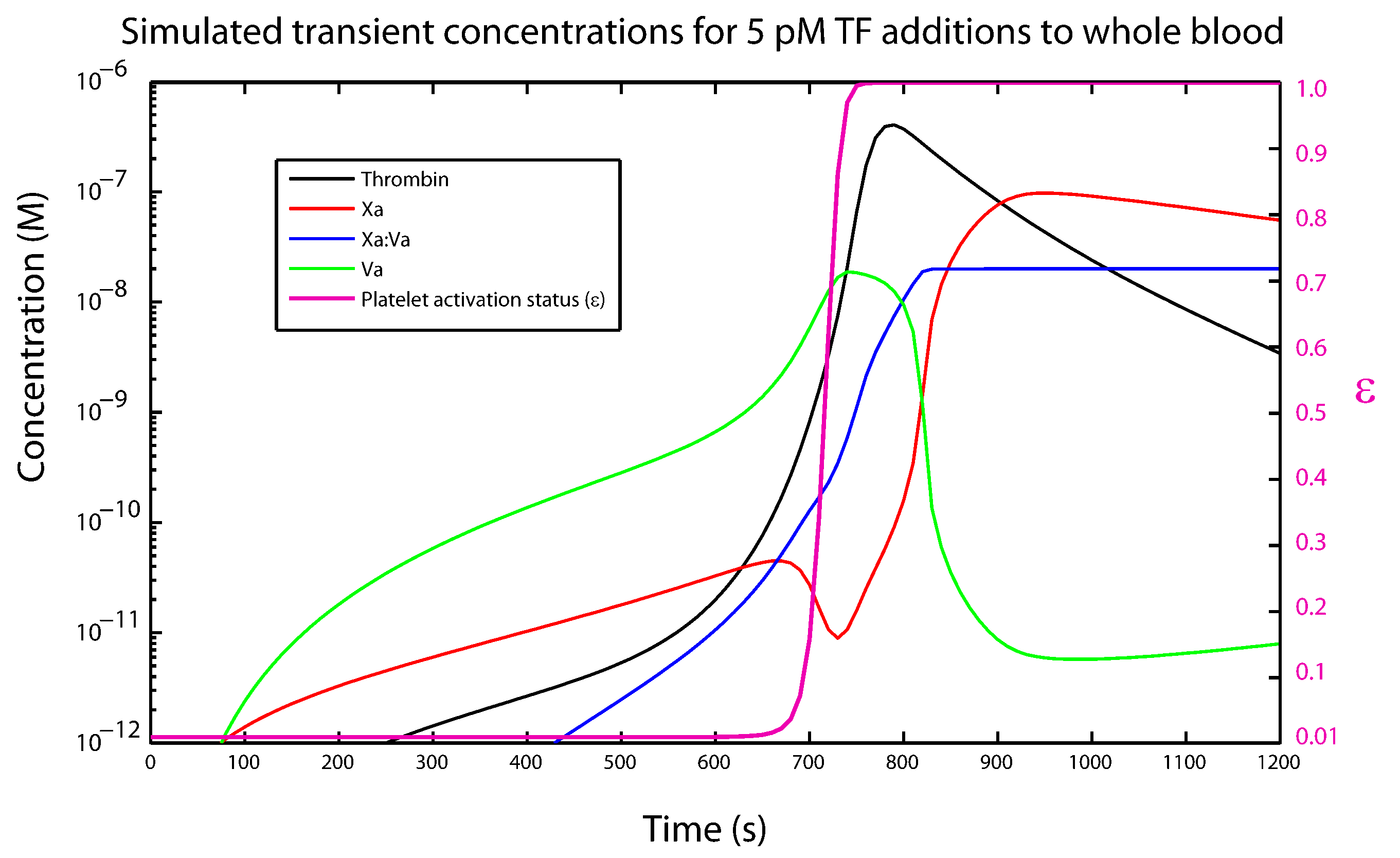


**Figure S2: Simulated time courses of selected species during coagulation with 5pM TF**

Shown above are the time courses of thrombin, Xa, Xa:Va, Va and the platelet’s activation state (ε) for coagulation triggered with 5pM TF in whole blood. Before initiation sufficient catalyst quantities are built up by the minute (subnanomolar levels) of thrombin being formed. Concentrations of all species increase steeply beyond initiation (and full platelet activation) during the propagation phase of the thrombogram. Note, the shift in equilibrium of Va towards Xa:Va following platelet activation. Thrombin concentrations eventually start decreasing because of destruction of the free enzyme by ATIII and consumption of limited reserves of prothrombin inspite of the presence of sufficient prothrombinase catalyst.

**Supplementary References:**

1. Bentele M, Lavrik I, Ulrich M, Stosser S, Heermann DW, et al. (2004) Mathematical modeling reveals threshold mechanism in CD95-induced apoptosis. Journal of Cell Biology 166: 839-851.

2. Sobol IM (2001) Global sensitivity indices for nonlinear mathematical models and their Monte Carlo estimates. Mathematics and Computers in Simulation 55: 271-280.

3. Saltelli A, Tarantola S, Chan KPS (1999) A quantitative model-independent method for global sensitivity analysis of model output. Technometrics 41: 39-56.
